# Activation of Nrf2 by Natural Bioactive Compounds: A Promising Approach for Stroke?

**DOI:** 10.3390/ijms21144875

**Published:** 2020-07-10

**Authors:** Agnese Gugliandolo, Placido Bramanti, Emanuela Mazzon

**Affiliations:** IRCCS Centro Neurolesi “Bonino Pulejo”, Via Provinciale Palermo, Contrada Casazza, 98124 Messina, Italy; agnese.gugliandolo@irccsme.it (A.G.); placido.bramanti@irccsme.it (P.B.)

**Keywords:** stroke, oxidative stress, natural compound, Nrf2, nutraceutics

## Abstract

Stroke represents one of the main causes of disability and death worldwide. The pathological subtypes of stroke are ischemic stroke, the most frequent, and hemorrhagic stroke. Nrf2 is a transcription factor that regulates redox homeostasis. In stress conditions, Nrf2 translocates inside the nucleus and induces the transcription of enzymes involved in counteracting oxidative stress, endobiotic and xenobiotic metabolism, regulators of inflammation, and others. Different natural compounds, including food and plant-derived components, were shown to be able to activate Nrf2, mediating an antioxidant response. Some of these compounds were tested in stroke experimental models showing several beneficial actions. In this review, we focused on the studies that evidenced the positive effects of natural bioactive compounds in stroke experimental models through the activation of Nrf2 pathway. Interestingly, different natural compounds can activate Nrf2 through multiple pathways, inducing a strong antioxidant response associated with the beneficial effects against stroke. According to several studies, the combination of different bioactive compounds can lead to a better neuroprotection. In conclusion, natural bioactive compounds may represent new therapeutic strategies against stroke.

## 1. Introduction

Stroke represents the second cause of death and one of the main causes of disability. Its incidence is increasing, representing a global health problem. The pathological subtypes of stroke are ischemic stroke and hemorrhagic stroke, that includes intracerebral hemorrhage (ICH) and subarachnoid hemorrhage (SAH) [1]. In particular, it was reported that 87% are ischemic stroke, 10% are represented by ICH, while only 3% are SAH [2]. Different risk factors are known to increase the risk for stroke, such as hypertension, high-risk diet, nutrition, obesity [1].

Nowadays, tissue plasminogen activator (tPA) represents the only therapeutic agent approved to treat patients with ischemic stroke and should be administered within 4.5 h from stroke onset. Its therapeutic actions are connected to thrombolysis and revascularization. However, tPA administration is associated also with adverse effects, because it can increase the risk of intracranial hemorrhage, edema, and it may exacerbate the brain damage, increasing blood brain barrier (BBB) disruption [3]. In case of ICH, the strategies aim to reduce bleeding [4]. tPA can be also used in hemorrhagic stroke to clear clotted blood from the brain. In hemorrhagic stroke, the mass effect caused by hematoma and the possible interruption of cerebrospinal fluid circulation in case of subarachnoid or intraventricular bleeding, causes an increase in intracranial pressure. In this case tPA can induce hematoma dissolution and restore cerebrospinal fluid circulation. However, it was shown that tPA can also induce inflammation [3].

Successful recanalization, that induce blood reflow, is the first purpose after the ischemic stroke. However, revascularization caused also complications, including ischemia/reperfusion (I/R) injury. The occurrence of I/R injury after the administration of a revascularization therapy can aggravate the situation because of the free radical damage. Indeed, free radicals can cause damage to subcellular components, including protein and DNA, and lipid peroxidation, causing cell death [5]. Also in the case of hemorrhagic stroke other than the primary damage caused by the hematoma mass effect, the second injury has a fundamental role, because of the onset of the pro-inflammatory cascade and the oxidative stress [6]. Oxidative stress, indicated as an alteration between reactive oxygen species (ROS) production and elimination, plays a main role in stroke. For this reason, counteracting oxidative stress could be considered as a potential therapeutic approach. In particular, ischemic brain is highly susceptible to oxidative injury because of the elevated oxygen (O_2_) consumption, the elevated amount of iron and unsaturated lipids, and the quite low endogenous antioxidant capacity [7]. Owing to its ability to counteract oxidative stress, the induction of Nuclear factor erythroid 2-related factor 2 (Nrf2) may protect the brain from the oxidative stress during stroke [8]. In particular, different natural compounds are able to induce Nrf2-promoting health benefits.

There is a great interest in natural compounds for the development of new therapeutic strategies against different pathologies. Indeed, natural products represent a source of different molecules. Since ancient times, natural compounds, and especially plant-derived ones, have been extensively used for the treatment of different diseases.

The advantages of the use of natural products are that they are already present in the human diet and for this reason their use may avoid the adverse effects caused by some synthetic drugs. Moreover, plants may be used as a direct source of therapeutic agents, or may be used as prototypes for the design of lead molecules. They possess also a more complex chemical structure and may possess a better tendency to interact with other molecules [9]. Natural products may be used as nutritional supplements. Indeed, normally the concentration of the bioactive components in food is not enough to reach the efficacious doses. On the contrary, the development of food supplements allows to administrate higher doses of the bioactive compounds. Moreover, given their safe profile they may be administrated also in the healthy population in order to prevent the onset of diseases. However, natural products may also be modified to be more adapt for the clinical use, in order to improve their selectively, their chemical and pharmacokinetic properties, such as their solubility or absorption [10].

In this review, we focused on the studies performed on in vivo and in vitro stroke models, that evaluated the protective effects of natural compounds through the activation of Nrf2 pathway. In order to select the studies, we performed a PubMed search using the keywords “stroke” and “Nrf2,” considering the experimental studies published from 2015 that evaluated in in vivo stroke models, both in vivo and in vitro stroke models or in microglia and astrocyte in vitro models the efficacy of the natural compounds showing the involvement of Nrf2.

## 2. Nrf2 and Its Regulation

Nrf2 is a transcription factor known to regulate the redox homeostasis in cells. Nrf2 is tightly regulated at different levels, including at transcription, by epigenetic modifications and after translation, leading to its activation during oxidative stress, inflammation, after several stimuli, such as growth factors, and allow to respond to different forms of stress [11]. Nrf2 modulates the expression of more than 200 genes. These genes present in the promoter region the so-called antioxidant response element (ARE). The genes regulated by Nrf2 encode for enzymes that participate in endobiotic and xenobiotic metabolism, oxidative stress and inflammatory responses, carbohydrate and lipid metabolism, and protein degradation [11].

Kelch- like ECH-associated protein 1 (Keap1) represents a negative regulator of Nrf2 [12]. In the cytoplasm, Keap1 creates a ubiquitin E3 ligase complex with Cullin3 that targets Nrf2 for polyubiquitination and fast proteasomal degradation [13,14]. In unstressed conditions, Nrf2 is ubiquitylated by Keap1 for proteasomal degradation, leading to Nrf2 short half-life and its presence in low amount. On the contrary, in stress conditions and exposure to electrophiles or ROS, cysteines in Keap1 are modified and it becomes inactive. Then, Nrf2 can rapidly translocate into the nucleus, where it can bind small musculoaponeurotic fibrosarcoma oncogene homologue (sMAF) proteins and induce the genes that contain ARE [14].

Nrf2–Keap1 axis exerts a protective effect in different disorders that show as main pathological mechanisms oxidative stress and inflammation, including cardiovascular disease and neurodegenerative disorders [15].

Nrf2 pathway is controlled at different levels. At first at the transcriptional level, Nrf2 is regulated by different transcription factors, including aryl hydrocarbon receptor (Ahr), a transcription factor involved in xenobiotic response, and nuclear factor kappa-light-chain-enhancer of activated B cells (NF-κB). A connection seems to exist between Nrf2 and NF-κB pathways [16,17]. How NF-κB regulates Nrf2 is complex and not fully clear, but some of the reported mechanisms are: the p65-mediated increase of nuclear Keap1, the competition for the binding to the transcriptional co-activator CBP (CREB-binding protein)-p300 complex, the p65-induced repression of transcription. However, in some cell types, p65 can induce the transcription of Nrf2 [17]. At post-transcriptional levels, different microRNAs (miRNAs) were discovered to be able to regulate Nrf2, such as miR-144, that was the first to be discovered. Another way to control Nrf2 is the regulation of the protein stability. Indeed, Nrf2 protein stability can be controlled by proteins that interfere with the interaction with Keap1. For example, p62 is reported to compete with Nrf2 for the binding with Keap1. In particular, it can cause Keap1 degradation stabilizing Nrf2. In addition also Keap-1 independent pathways can modulate Nrf2 signaling. Glycogen synthase kinase-3β (GSK-3β) can control Nrf2 protein stability. GSK-3β can phosphorylate different residues of Nrf2. When phosphorylated Nrf2 can bind β-transducin repeat-containing protein (βTrCP), a scaffold protein that links Nrf2 with a ubiquitination complex, causing Nrf2 degradation. However, also other kinases can modulate Nrf2 activity, including protein kinase C (PKC), phosphoinositide 3-kinases (PI3K), and mitogen-activated protein kinase (MAPK) [18].

Nrf2 is subjected to a regulation also at the nuclear level. Here the BTB domain and CNC homolog 1 (BACH1) heterodimerizes with MAF, binds ARE in order to suppress the transcription of these genes. However, in oxidative stress conditions, BACH1 is phosphorylated and expelled into the cytoplasm, so that MAF can bind Nrf2 and induce the transcription of the target genes [19]. Among the modulator of Nrf2 in the nucleus, also Src subfamily A members Fyn, Src, Yes, and Fgr were found, that through the phosphorylation of Nrf2 caused its nuclear export and degradation [20].

Different nutrients present in the human diet and plant-derived compounds show health promoting effects that are exerted, at least in part, by Nrf2 pathway. Among these ones, we can find isothiocyanates found in cruciferous vegetables, organosulfur compounds, polyphenols, and isoflavones that have been found to be Nrf2 activators [21,22].

## 3. Natural Compounds as Modulator of Nrf2 Pathway in Stroke Animal Models

Experimental models are necessary to evaluate both the neuropathological mechanisms of stroke and to find novel treatments. The majority of stroke models were developed in mice and rats. Their advantages are the easy accessibility, the lower cost, the replicability, the similarities to humans in cerebrovascular structure, even if obviously differences exist between these animals and humans in different aspects of brain structure and functions [23,24]. Given that ischemic stroke represents the most frequent form of stroke, it is not surprising that the majority of studies on new therapeutic approaches were performed on ischemic stroke models. Ischemic stroke in humans is generally caused by the occlusion of the middle cerebral artery (MCA) and for this reason MCA occlusion (MCAO) model represents the most used in experimental studies [23,25]. Regarding hemorrhage models, also in this case most often used animal models are rat and rabbit species and the experimental SAH was induced by intracisternal blood injection and endovascular vessel perforation [26].

Different natural compounds, present in the diet, as food or as dietary supplements, or other plant-derived compounds were tested for their potential beneficial effects against stroke owing to their capacity to activate Nrf2. In the next paragraphs, the studies showing protective effects of natural bioactive compounds in experimental stroke models through the involvement of Nrf2 signaling are discussed.

### 3.1. Tea Bioactive Compounds

**Figure 1:**
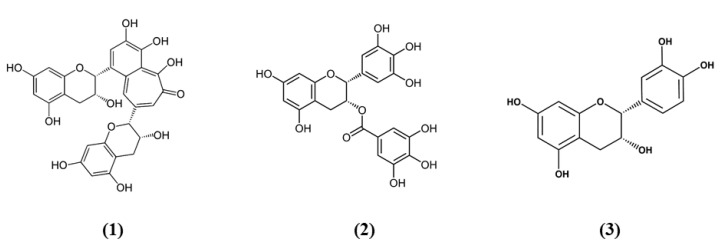
Chemical structure of (**1**) theaflavin, (**2**) (-)-epigallocatechin-3-gallate, and (**3**) (-)-epicatechin.

Tea is one of the most widely used beverage worldwide and it is a rich source of bioactive compounds shown to exert different health benefits. In particular, polyphenols represent the principal bioactive molecules found in teas. Among polyphenols, catechins are the main ones in green tea, while the black tea contains theaflavins [27]. Li et al. evidenced that theaflavin (C_29_H_24_O_12_; CAS number 4670-05-7; Figure 1 (1)) exerted beneficial effects both in in vivo and in vitro models of I/R abolishing miRNA-128-3p-induced Nrf2 suppression and thus decreasing oxidative stress [28]. In particular, in rats subjected to MCAO, theaflavin administration lowered infarct volume and neuronal damage, and ameliorated neurological abilities. In vitro, in neural stem cells (NSCs) exposed to oxygen glucose deprivation (OGD), theaflavin pretreatment enhanced proliferation and reduced apoptosis. Theaflavin also dose-dependently reduced ROS and malondialdehyde (MDA), while superoxide dismutase (SOD) and glutathione peroxidase (GPx) activities were elevated in rat brain tissue and in NSCs. These enzymes have an important role in counteracting oxidative stress: SOD catalyzes the reaction of dismutation of the superoxide radical leading to the formation of O_2_ and hydrogen peroxide (H_2_O_2_), while GPx eliminates H_2_O_2_ forming water and O_2_, through the oxidation of glutathione (GSH). The antioxidant effects of theaflavin may be due to its capacity to increase dose-dependently Nrf2 expression reducing miRNA-128-3p levels in vivo and in vitro [28]. It is important to mention that MDA shows some limits in order to be considered as a marker of oxidative stress. Indeed, because of its high reactivity, its tendency to cross-react and the limitations of the methods used to measure its levels, it was suggested that MDA cannot be considered as a reliable oxidative stress marker [29].

Also the potential protective effects of (-)-epigallocatechin-3-gallate (EGCG) (C_22_H_18_O_11_; CAS number 989-51-5; Figure 1 (2)), another catechin found in green tea, have been evaluated in stroke. In particular, Bai et al. evaluated EGCG effects on angiogenesis after stroke and the involvement of Nrf2, using a MAPK/extracellular signal-related kinase (MAPK/ERK) inhibitor, given that EGCG was reported to induce Nrf2-mediated antioxidant action through the MAPK/ERK pathway. The results obtained showed that EGCG treatment improved neurologic outcome, reduced infarct volume, increased angiogenesis in association with an upregulation of vascular endothelial growth factor receptor 2 (VEGFR2) signaling pathway and increased Nrf2 nuclear levels. EGCG enhanced also SOD1-positive cells, while endoplasmic reticulum stress markers GRP78, CHOP, and Caspase 12 levels were decreased. However, MAPK/ERK inhibitor abolished the protective effects exerted by EGCG and reduced nuclear Nrf2 expression, and the number of SOD1 positive cells in the peri-infarction area [30]. Leonardo et al. evaluated the effects of another cathechin, (-)-epicatechin (EC) (C_15_H_14_O_6_; CAS number 490-46-0; Figure 1 (3)), against stroke in aging wildtype (WT) and Nrf2^−/−^ mice. 12-month-old WT mice pretreated with EC before permanent MCAO (pMCAO) showed a reduction in infarct volume and ameliorated score in removing adhesive tape compared with controls. However, EC treatment in aging Nrf2^−/−^ mice showed a trend for the reduction of infarct volume, but not statistically significant. This result indicated that Nrf2 modulated protection in the aging phenotype at least in part, but that with aging, the action of Nrf2 is weak. However, EC treatment did not alter microglia activation, vascular permeability or spontaneous hemorrhage [31].

The weakened effect of Nrf2 with aging is important taking into account that it is reported the age-related decline in Nrf2 activation and consequently gene expression of its target genes. Indeed, both older humans and animals showed decreased Nrf2 nuclear levels and Nrf2 activation. Nrf2 decrease was suggested to be due to the enhanced levels of its negative regulators and a direct decrease in Nrf2 levels. In particular, the mechanisms that participate in the decrease of Nrf2 with age are: an increase in Keap1 levels, the decrease of p62 levels, modulation by GSK-3β/β-TrCP, increased levels of BACH1, and modulation by miRNAs [32,33,34]. For this reason, compounds that may induce its activity could at least in part compensate for the reduced activation. Moreover, it would be helpful also to administer them with other compounds that reduce the levels of Nrf2 negative regulators. This argument is particularly important in the context of stroke, where the patients affected are commonly elderly. Moreover, it may be helpful to start a supplementation with Nrf2 activators as a preventive treatment, in particular in high risk populations. Then, both pharmaceutical and dietary modulation that increase Nrf2 activation during aging and under stress conditions may be helpful. The effects of EC were evaluated also in vitro in astrocytes obtained from WT and Nrf2^−/−^ mice treated with hemoglobin to create an ICH model. EC upregulated Nrf2 and SOD1 only in WT astrocytes, while it reduced phosphorylated c-Jun N-terminal kinases (JNK), nuclear JNK, and activator protein 1 (AP-1) in both WT and Nrf2^−/−^ astrocytes [35].

It is important to notice that plasma concentration of tea catechins after oral administration ranged in the sub- or low-µM, a dose that is lower compared to the doses that show efficacy [36,37]. The catechin concentrations in green tea ranged between about 3500 and 4500 mg/L, and studies suggested that low percentages of the orally administered doses were found in the plasma of animals and human subjects [36,37]. The poor bioavailability is due to the poor stability in the gut, poor absorption, first-pass metabolism, and low accumulation. For this reason, new methods that improve catechin bioavailability need to be developed such as nanoparticle delivery systems or other carriers, or the co-administration with other compounds [36].

### 3.2. Citrus Bioactive Compounds

**Figure 2:**
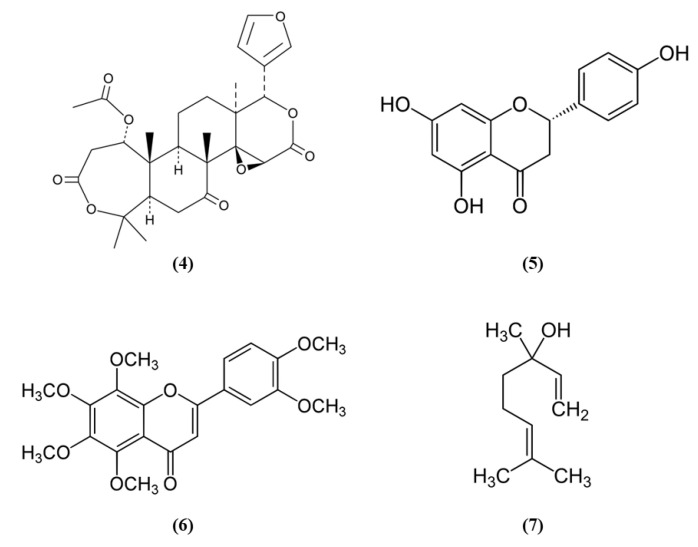
Chemical structure of (**4**) nomilin, (**5**) naringenin, (**6**) nobiletin, and (**7**) linalool.

Citrus fruits represent a good source of dietary antioxidants with health promoting effects. Among the phytochemicals, different vitamins, flavonoids, coumarins, terpenoids, and others are present in the citrus fruits [38]. Nomilin (Nom) (C_28_H_34_O_9_; CAS number 1063-77-0; Figure 2 (4)), showed beneficial effects in vitro in SH-SY5Y cells exposed to OGD and in an experimental model of cerebral I/R. In vitro, Nom treatment dose-dependently reduced cell death and ROS level, through the increase in nuclear Nrf2 together with its downstream enzyme NAD(P)H quinone oxidoreductase 1 (NQO1). On the contrary, Nrf2 knockdown suppressed Nom protective actions. In vivo, Nom treatment reduced infarct volume, brain edema, and neurologic score. In addition, Nom attenuated BBB disruption in MCAO rats, owing to the inhibition of the tight junction proteins zonulin 1 (ZO-1) and occludin-5 loss. The treatment with Nom reduced apoptosis and ameliorated the neuronal morphology. Moreover, Nom treatment reduced oxidative stress, as evidenced by the augmented activity of SOD, catalase (CAT), and GPx and the reduction of MDA levels. CAT is another important antioxidant enzyme, that catalyzes the elimination of H_2_O_2_ forming water and O_2_. Then it is important to notice that H_2_O_2_ formed by SOD can be eliminated by CAT and GPx. Also in vivo Nom was able to increase the expressions of nuclear Nrf2 and NQO1 [39]. It is important to notice that ROS evaluation was performed using dichlorofluorescin-diacetate (DCFH-DA). Although DCFH-DA is commonly employed for the measurement of H_2_O_2_ and oxidative stress, this method shows several limits and can cause artifacts. Specifically, DCFH-DA does not represent a direct evaluation of H_2_O_2_, because DCFH-DA oxidation can also be mediated by redox-active metals when O_2_ or H_2_O_2_ are present, but also cytochrome c is able to cause the oxidation of DCFH-DA. In addition, one-electron-oxidizing species can induce the oxidation of DCFH-DA to DCF and the intermediate radical is able to react with O_2_ forming superoxide, and in turn its dismutation forms H_2_O_2_ causing the erroneous amplification of the signal [40]. All these limitations may cause a misinterpretation of the results.

Another phytocompound found in citrus fruit is the flavanone naringenin (Nar) (C_15_H_12_O_5_; CAS number 480-41-1; Figure 2 (5)). Wang et al. found that Nar induced cell proliferation, reduced apoptosis, and prevented the reduction of mitochondrial membrane potential in OGD-exposed cortical neurons. Moreover, Nar increased mRNA levels of Nrf2, Keap1, heme oxygenase 1 (HO-1), and NQO1, while Nrf2 silencing reverted these beneficial effects. HO-1 catalyzes the degradation of heme leading to the formation of carbon monoxide, iron, and biliverdin. Tissue damage induces the release of heme that can amplify the injury, for this reason HO-1 has an important protective role, but it also takes part in important cellular pathways. Nar can regulate Nrf2 localization, increasing cytoplasmic Nrf2 and reducing nuclear Nrf2 both in vitro and in vivo. In vivo, Nar alleviated brain edema and apoptosis and improved neurological score. These results suggested that Nar may exert beneficial effects in stroke through the involvement of Nrf2 signaling pathway [41].

However, not only citrus fruits, but also its peel is rich in antioxidant compounds and one of these is nobiletin (Nob) (C_21_H_22_O_8_; CAS number 478-01-3; Figure 2 (6)) [42]. Zhang et al. evaluated the protective effects exerted by different Nob concentrations in rats subjected to pMCAO. High Nob doses reduced neurological deficits, brain edema, and infarct volume. In addition, the highest Nob dose exerted a strong antioxidant action, increasing nuclear Nrf2, HO-1, SOD1 activity, and GSH and reduced MDA levels. Nob exerted also an anti-inflammatory action at high dosage, as demonstrated by the increased expression of NF-κB in the cytoplasm and its decreased expression in the nucleus. Moreover, only the highest concentration of Nob was able to decrease metalloproteinase (MMP) 9. The results then indicated that Nob exerted antioxidant actions through Nrf2 pathways, but also anti-inflammatory effects were involved in its beneficial action [43]. It must be noticed that the determination of GSH levels in tissues is quite difficult and requires different steps and accuracy. Several methods were used, leading to different finding that can lead to erroneous interpretation of the results. However, previously a method was described that was easy and sensitive [44].

Linalool (C_10_H_18_O; CAS number 78-70-6; Figure 2 (7)) is another compound found both in citrus peel and citrus essential oils [42,45]. Barrera-Sandoval et al. evaluated the effects of intranasal administration of linalool against ischemia. The intranasal administration may have the advantage of a more rapid release at the level of the central nervous system. Linalool administration reduced infarct volume after 24 h and a week and improved neurological and motor functions. Moreover, linalool was able to decrease microgliosis, cyclooxygenase 2 (COX2), and Nrf2 in hippocampus after a month. In particular, Nrf2 showed a different distribution: Nrf2 showed a punctate pattern in sham animals, while it appeared aggregated in activated microglia in MCAO rats. In vitro, in astrocyte and microglial cultures exposed to glutamate, the main neurotransmitter involved in ischemia, linalool reduced pro-inflammatory markers, and caused Nrf2 subcellular redistribution [46].

However, it is important to take into account the bioavailability of these compounds. Nom showed a low bioavailability after oral administration, that is nearly 4% [47]. Nar was found to be rapidly absorbed after oral administration and it was found in plasma already 20 min after administration, but the bioavailability was low probably because of first pass metabolism [48]. However, the consumption of a diet with a high content of vegetables increases its plasma concentrations, even if a high variability among individuals was recorded [49]. Also for Nob the blood concentrations were found to be in the µM range. After absorption, Nob is distributed into the different organs and in some of them, including brain, significant levels were detected [50]. Interestingly, both Nar and Nob were also found in the brain [51]. In particular, in rats it was demonstrated that after oral administration of Nob, its brain concentrations were about triplicate compared to plasma concentrations and in brain Nob was detectable for 24 h compared to 9 h in plasma indicating a lower elimination at brain level [52]. However, also in this case, as already said for tea bioactive compounds, the development of methods to increase bioavailability may be helpful [52]. Also for linalool, that is characterized by poor oral bioavailability, nanostructured lipid carriers were suggested [53].

### 3.3. Spice Bioactive Compounds

**Figure 3:**
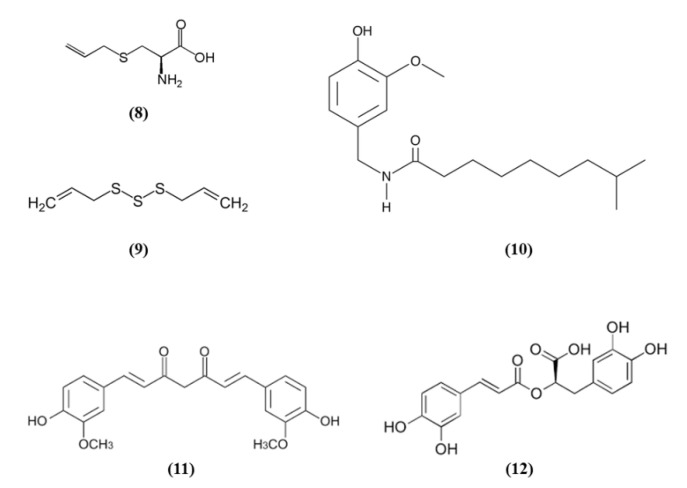
Chemical structure of (**8**) S-allyl cysteine, (**9**) diallyl trisulfide, (**10**) dihydrocapsaicin, (**11**) curcumin, and (**12**) rosmarinic acid.

Spices were used since ancient times for culinary and medicinal purposes. In particular, the bioactive compounds present in spices can exert antioxidant and health promoting effects. Spices such as garlic, turmeric, chili peppers, and rosemary are among the most used spice worldwide and they contain S-allyl cysteine (SAC) (C_6_H_11_NO_2_S; CAS number 21593-77-1; Figure 3 (8)) and diallyl trisulfide (DATS) (C_6_H_10_S_3_; CAS number 2050-87-5; Figure 3 (9)), dihydrocapsaicin (DHC) (C_18_H_29_NO_3_; CAS number 19408-84-5; Figure 3 (10)), curcumin (C_21_H_20_O_6_; CAS number 458-37-7; Figure 3 (11)), and rosmarinic acid (RA) (C_18_H_16_O_8_; CAS number 20283-92-5; Figure 3 (12)), respectively [54].

SAC was able to exert cytoprotective effects, inducing Nrf2 and its downstream targets and suppressing JNK and p38 phosphorylation in cortical neurons exposed to OGD. Interestingly, the knockdown of Nrf2 abolished the SAC beneficial effects. SAC administration to mice subjected to MCAO caused the increase in Nrf2 and of its targets in cerebral cortex and hippocampus and decreased phosphorylated JNK and p38 levels. Moreover, SAC administration decreased infarct volume and neurological score and ameliorated neuronal survival only in WT mice, not in Nrf2^−/−^ animals, indicating that Nrf2 pathway is involved in SAC protective effects [55]. Silva-Islas et al. showed that DATS improved the motor function, reduced infarct volume and the brain injury in the striatum and cortex of MCAO rats. DATS treatment reduced MMP9 levels, considered as an indirect marker of BBB injury. In rats receiving DATS decreased MDA levels were reported. Interestingly, Nrf2 induction increased in the cortex as well as SOD1 in the nucleus, SOD2 and glutathione S-transferase (GST) at both striatum and cortex levels [56].

DHC effects on BBB disruption in cerebral I/R models were evaluated by Janyou et al. [57]. DHC decreased neurological deficit scores, infarct area, morphological modifications in the neuronal cells associated with a reduction of apoptosis. DHC at the highest dose also reduced the BBB damage and water content, owing to the increase of the tight junction proteins occludin and claudin. DHC also reduced the I/R-induced BBB ultrastructure modifications, astrocytic swelling around the endothelial cells, modifications of the vascular lumen and the numbers of microvilli. DHC also increased the expression of Nrf2 and NQO1 that exerted an antioxidant function. Indeed, the activities of SOD and GPx were increased, while MDA and nitric oxide (NO) levels were decreased. Moreover, DHC treatment also decreased NADPH oxidase (NOX2, NOX4), p65 subunit of NF-ĸB, and MMP9 levels. However, the lowest dose tested was not efficacious [57].

Also, different doses of RA were able to attenuate ischemic brain injury as demonstrated by the improvement of neurological function, the reduced infarct volume, and the decreased apoptosis. RA exerted also antioxidant effects, as demonstrated by the increased cortical SOD activity, HO-1 and Nrf2 expression, and the reduced MDA amounts. Interestingly, a HO-1 inhibitor abolished the positive actions of RA on apoptosis. The inhibitor of PI3K/Akt signaling pathway abolished Akt phosphorylation, together with Nrf2 and HO-1 expressions. These results suggested that RA was able to counteract oxidative stress and apoptosis inducing Nrf2/HO-1 pathway through the PI3K/Akt signaling [58].

Curcumin, not only decreased neurological deficits, brain edema, and infarct volume, but it was also able to reverse the MCAO-induced BBB disruption. Curcumin protection was due to the decrease of NF-κB expression and the upregulation of Nrf2 [59]. Similarly, hexahydrocurcumin (HHC), that represents one of the main metabolites of curcumin obtained from phase I metabolism, reduced neurological deficits, neuron degeneration, and infarct volume. In line with the previous work on curcumin, also HHC exerted anti-oxidant and anti-inflammatory actions, as shown by the reduction of MDA, NO, NF-κB, and COX-2 expression in the I/R group and the increase in nuclear Nrf2, HO-1, SOD, GSH, and GPx levels. Moreover, the HHC treatment also significantly decreased apoptosis. Given these results, HHC was suggested to exert a protective action against oxidative stress, inflammation, and apoptosis in stroke [60].

### 3.4. Fruit Bioactive Compounds

**Figure 4:**
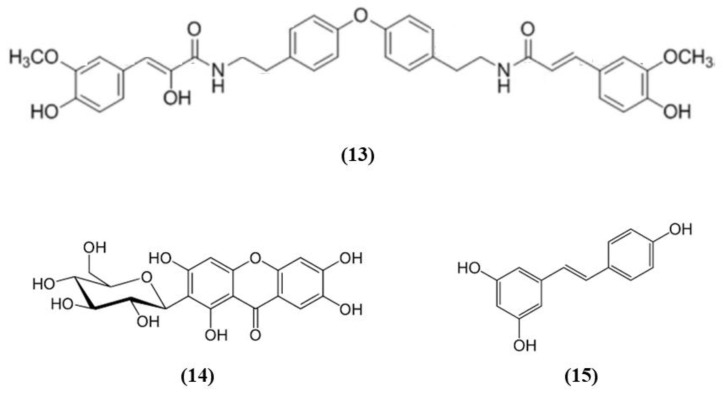
Chemical structure of (**13**) lyciumamide A, (**14**) mangiferin, and (**15**) resveratrol.

Some fruits, including exotic fruits, gained the name of “superfruit” for their great beneficial effects. Among these ones also Goji berry, that contains different bioactive compounds, including flavonols, monoterpenes, phenolic acids, and lyciumamides [61]. Lyciumamide A (LyA) (C_36_H_36_N_2_O_8_; Figure 4 (13)), derived from *Lycium barbarum* fruits, better known as Goji berries, is a dimer of phenolic amide. LyA exerted protective effects in the in vivo model of MCAO, but also in vitro in SH-SY5Y cells exposed to OGD. In the animals subjected to MCAO, LyA administration ameliorated neurological deficits, decreased infarct volume, and neuronal damage. LyA showed antioxidant activity, as demonstrated by the increase of nuclear Nrf2 levels, cytoplasmic HO-1 expression, cortical SOD and GPx activities and by the reduction of MDA level compared to the MCAO group. In vitro LyA pretreatment reduced apoptosis and exerted an antioxidant action increasing Nrf2 in the nucleus and HO-1 cytoplasmic levels. Interestingly, knocking down Nrf2 or HO-1 abolished LyA beneficial effects, indicating that LyA exerted anti-oxidative effects through the induction of the Nrf2/HO-1 pathway. Given the known role of PKC in Nrf-2 induction, also its involvement was evaluated. LyA increased the levels of pPKCε and knockdown of PKCε suppressed the activation of Nrf2/HO-1 and the beneficial actions exerted by LyA. All together these results indicated that LyA protective effects in stroke were due to the PKCε/Nrf2/HO-1 pathway [62].

Mangiferin (C_19_H_18_O_11_; CAS number 4773-96-0; Figure 4 (14)) is a glucosyl xanthone present in some fruits, such as mango and papaya. Mangiferin dose-dependently improved neurological score, infarct volume, and edema in rats subjected to cerebral I/R injury. However, the lowest dose tested was not efficacious in regards of infarct volume. Mangiferin was able to exert both anti-oxidant and anti-inflammatory effects, reducing MDA, interleukin (IL)-1β, and tumor necrosis factor (TNF)-α, and increasing the brain SOD and GSH activities and IL-10 levels in cerebral I/R rats. In addition, mangiferin increased Nrf2 in the nucleus and HO-1. These findings indicated that mangiferin exerted a beneficial action in the cerebral I/R injury that is mediated by the Nrf2/HO-1 signaling [63].

Also grape is rich in different bioactive compounds able to exert beneficial effects, and resveratrol (C_14_H_12_O_3_; CAS number 501-36-0; Figure 4 (15)) is one of the most studied [64]. Narayanan et al. evaluated the involvement of Nrf2 in resveratrol preconditioning in astrocyte cultures and in an experimental stroke model. Resveratrol preconditioning decreased infarct volume compared with mice subjected to MCAO treated with vehicle, but not in Nrf2^−/−^ animals. The authors determined Nrf2 involvement in resveratrol-induced protection and Nrf2 activation in astrocytes given that these cells showed a great Nrf2 amount. Nuclear Nrf2 was activated 48 h after resveratrol preconditioning in astrocytes, increasing NQO1. In addition, resveratrol preconditioning caused uncoupling in both WT and Nrf2^−/−^ mitochondria. However, Nrf2^−/−^ mitochondria showed an innate respiratory dysfunction. Interestingly, resveratrol preconditioning increased the expression of NQO1 in WT astrocytes, but not in Nrf2^−/−^ ones [65].

### 3.5. Other Dietary Bioactive Compounds

Polyphenols are characterized by the presence of aromatic rings and more than one hydroxyl group. The food rich in polyphenols shows several health-promoting effects [66].

**Figure 5:**
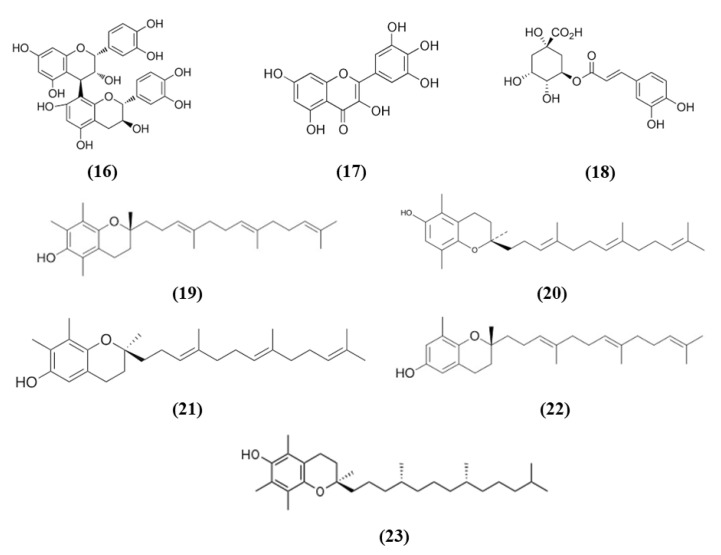
Chemical structure of (**16**) procyanidin B2, (**17**) myricetin, (**18**) chlorogenic acid, (**19**) α-tocotrienol, (**20**) β-tocotrienol, (**21**) γ-tocotrienol, (**22**) δ-tocotrienol, and (**23**) α-tocopherol.

Procyanidins, polyphenols present in different plant food, are formed by (+)-catechin and/or EC units. Procyanidin B are the predominant ones in several foods, including berries, cereals, nuts, legumes, chocolate, and wines [67]. In particular, procyanidin B2 (PB) (C_30_H_26_O_12_; CAS number 29106-49-8; Figure 5 (16)) is mainly present in cocoa and fruits such as apples and grapes and it is formed by two flavan-3-ol EC molecules. Wu et al. evaluated its effects on BBB disruption induced by ischemic stroke, studying its mechanism of action. Treatment with different PB doses reduced infarct volume together with brain edema, however only the highest one was used for further experiments given that it exerted the best effects. Interestingly, PB treatment improved neurological deficits after MCAO. These protective effects were associated with the prevention of BBB damage, attenuating tight junction degradation as demonstrated by the rescue of ZO-1. PB also counteracted oxidative stress as demonstrated by the reduction of mitochondrial depolarization, ROS and MDA levels in the ischemic brain and the increase of SOD and CAT activities. The PB antioxidant action was associated with the restoration of Nrf2 nuclear translocation in the ischemic brain, with the consequent elevation of HO-1, GSTα, and NQO1 levels. The results indicated that PB exerted neuroprotective effects and inhibited BBB damage in an experimental model of cerebral ischemia through Nrf2 pathway [68].

Also myricetin exerted protective effects through the Nrf2 pathway. Myricetin (C_15_H_10_O_8_; CAS number 529-44-2; Figure 5 (17)) is a common plant-derived flavonoid known for its nutraceutical value. It is very common in fruits, berries, vegetables, and in teas and wines [61,69]. Pre-treatment with myricetin attenuated cytotoxicity, mitochondrial depolarization and ROS levels in SH-SY5Y cells exposed to OGD. In the MCAO rat model, treatment with myricetin reduced infarction volume, neuronal loss, and improved neurological function. Moreover, myricetin also decreased oxidative stress, with a reduction in ROS production and MDA levels, while increasing CAT and SOD activity. Also, in mitochondria, myricetin treatment prevented the reduction of mitochondrial potential and mitochondrial ATP and the increase of mitochondrial MDA and ROS levels. The antioxidant effect of myricetin treatment was associated with the increased nuclear Nrf2 levels in the ischemic brains, but also in normal ones, but cytosolic Nrf2 protein levels were not influenced. As a consequence also HO-1 levels increased [70].

Chlorogenic acid (CGA, 5-O-caffeoylquinic acid) (C_16_H_18_O_9_; CAS number 906-33-2; Figure 5 (18)), is another polyphenol present in *Coffea canephora, Coffea arabica* L., and *Mate (Ilex paraguariensis A. StHil.*). Liu et al. evidenced that CGA administration reduced infarct volume, brain edema, and neurological deficits in a dose-dependent manner in I/R model. Moreover, CGA promoted brain-derived neurotrophic factor (BDNF) and nerve growth factor (NGF) expression and ameliorated the I/R-induced apoptosis and morphological hippocampal neuron damage. Moreover, CGA attenuated oxidative stress, as a consequence of increased SOD activity and GSH level, and the reduction in ROS and MDA. Interestingly, CGA rescued the I/R caused reduction of Nrf2 pathway and induced Nrf2 expression, together with the expression of the antioxidant enzymes NQO1 and HO-1. Moreover, Nrf2 pathway inhibitor suppressed CGA effects, suggesting that CGA neuroprotection is mediated by Nrf2 pathway [71].

Vitamin E, that includes tocopherols and tocotrienols, is found in some edible oils as almond, peanut, olive, and others [72]. A particularly interesting work evidenced that Tocovid, containing α-tocotrienol (C_29_H_44_O_2_; CAS number 58864-81-6; Figure 5 (19)), β-tocotrienol (C_28_H_42_O_2_; CAS number 490-23-3; Figure 5 (20)), γ-tocotrienol (C_28_H_42_O_2_; CAS number 14101-61-2; Figure 5 (21)), δ-tocotrienol (C_27_H_40_O_2_; CAS number 25612-59-3; Figure 5 (22)), and α-tocopherol (C_29_H_50_O_2_; CAS number 10191-41-0; Figure 5 (23)) was able to exert protective effects against ischemic stroke, ameliorating motor function and reducing infarct volume. The protective effects of Tocovid were associated to its antioxidant action, as demonstrated by the decreased number of 4-hydroxynonenal (4-HNE), nitrotyrosine, and 8-hydroxy-2′-deoxyguanosine (8-OHdG) positive cells, of advanced glycation end products, while Nrf2 increased in neurons, but also in microglia and astrocytes and endothelial cells in the ischemic area. The pretreatment with tocovid also decreased the ratio oxidized glutathione (GSSG)/GSH, in association with the increase of MRP1 levels, a major GSSG clearing system. Tocovid administration reduced also cleaved caspase-3 and LC3-II in the tMCAO group [73].

**Figure 6:**
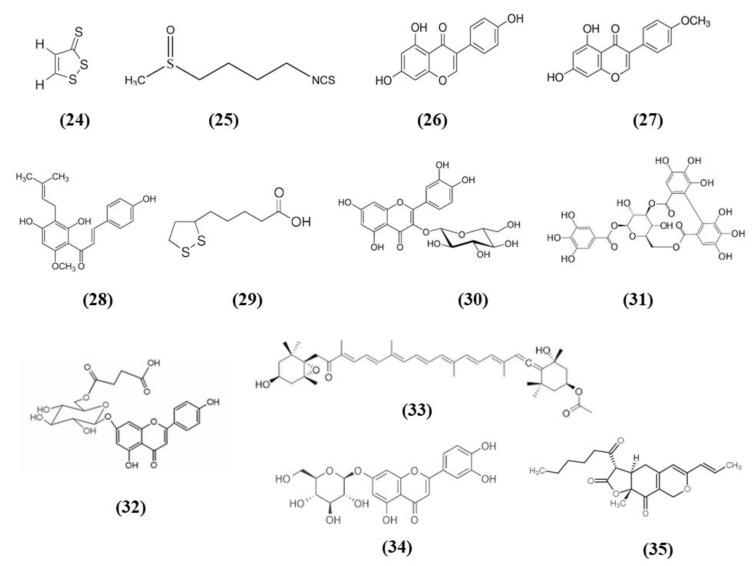
Chemical structure of (**24**) 3H-1,2-dithiole-3-thione, (**25**) sulforaphane, (**26**) genistein, (**27**) biochanin A, (**28**) xanthohumol, (**29**) α-lipoic acid, (**30**) isoquercetin, (**31**) corilagin, (**32**) 6″-O-succinylapigenin, (**33**) fucoxanthin, (**34**) luteoloside, and (**35**) monascin.

Cruciferous vegetables are a source of different bioactive compounds, such as dithiolethiones, whose simplest member is 3H-1,2-dithiole-3-thione (D3T) (C_3_H_2_S_3_; CAS number 534-25-8; Figure 6 (24)). Kuo et al. showed that D3T reduced infarct size and cerebral edema, ameliorated neurological deficits, and increased survival rate in a model of ischemic stroke. Moreover, D3T inhibits BBB disruption together with a reduction of MMP 9 and suppressed microglia activation. This data explained the reduced peripheral immune cell infiltration. Indeed, activated microglia is associated with chemokine production and peripheral immune cell infiltration. D3T induced Nrf2 expression in the brain of ischemic animals. Interestingly, the D3T protection in ischemic stroke was abolished in Nrf2^−/−^ animals. The authors investigated in vitro whether Nrf2 had a role in the inhibition of microglia activation mediated by D3T. They showed that Nrf2 induction exerted by D3T is necessary for the suppression of microglia activation. Indeed, D3T decreased the levels of several inflammatory markers in WT primary microglia cells treated with lipopolysaccharide (LPS), but not in Nrf2^−/−^ microglia. Also HO-1 was necessary for D3T neuroprotective effects, indeed its inhibition abolished D3T beneficial effects [74].

Another compound present in cruciferous vegetables is sulforaphane (C_6_H_11_NOS_2_; CAS number 4478-93-7; Figure 6 (25)), that was shown to be able to activate Nrf2 and to enhance hematoma clearance. Using microglia to model red blood cell clearance, sulforaphane exerted antioxidant action, and increased red blood cell phagocytosis. In particular, Nrf2 played a main role in these effects. In an in vivo autologous blood injection ICH model, sulforaphane induced CD36 expression and ameliorated hematoma clearance in rats and WT mice, but no effects were reported in Nrf2^−/−^ mice [75].

Phytoestrogens are compounds derived from plant with a structure similar to endogenous estradiol. They possess different beneficial actions. Among the main phytoestrogens in the form of isoflavones there are also genistein (C_15_H_10_O_5_; CAS number 446-72-0; Figure 6 (26)) and biochanin A (C_16_H_12_O_5_; CAS number 491-80-5; Figure 6 (27)) contained in soybeans [76], that were tested in stroke models. In particular, genistein was administered to ovariectomized rats subjected to MCAO, because postmenopausal women show a more prominent risk of stroke and estrogen replacement therapy seems to have protective effects. Genistein ameliorated neurological function, decreased infarct volume in association with a reduced neuronal damage in the ischemic penumbra. The neuroprotective effects of genistein were associated with its antioxidant action exerted through the increase of Nrf2 and NQO1 leading to the reduction of ROS and cleaved-caspase3 levels in ovariectomized rats [77].

Similarly to genistein, also biochanin A ameliorated neurological function, reduced infarct volume and brain edema dose-dependently. Also in this case the beneficial actions of the pre-treatment with biochanin A were associated with its antioxidant actions, that was mediated by Nrf2, that translocate to the nucleus. As a consequence, HO-1 increased together with SOD and GPx activities while the production of MDA decreased. However, biochanin A exerted also anti-inflammatory effects inhibiting dose-dependently NF-kB acti¬vation in ischemic brain injury [78].

Jiao et al. tested xanthohumol (XN) (C_21_H_22_O_5_; CAS number 6754-58-1; Figure 6 (28)), a flavonoid extracted from *Humulus lupulus*, that is hops, in MCAO and OGD models. In vivo, XN reduced infarct size, neuronal damage, and apoptosis, as well as improved survival rate and neurobehavioral test score. Moreover, XN was able to exert an antioxidant action, as shown by the reduction of 4-HNE and 8-OHdG positive cells, MDA level, and the ratio GSSG/GSH, while CAT and SOD were significantly increased. Also in vitro, XN reduced neuronal apoptosis owing to the inhibition of oxidative stress, indeed XN pretreatment improved mitochondrial membrane potential, reduced ROS, GSSG/GSH ratio, and MDA levels, increased CAT and SOD activities. Moreover, both in vivo and in vitro XN reduced p38 phosphorylation and induce the activation of nuclear Nrf2. As a consequence of Nrf2 activation, also HO-1 levels increased after XN treatment, both in vivo and in vitro [79].

Alpha-lipoic acid (α-LA) (C_8_H_14_O_2_S_2_; CAS number 1077-28-7; Figure 6 (29)) is an organosulfur compound present in plants, but also produced by animals and humans. It is found in mitochondria where it takes part in different chemical reactions in the Krebs cycle, being a cofactor for pyruvate dehydrogenase and α-ketoglutarate dehydrogenase. Even if it can be produced by the human body in low quantity, it is not enough for the energy requirement of the cells. For this reason, it is obtained from diet, in particular from meat and vegetables, and also from fruits. α-LA is found in red meat, spinach, tomatoes, broccoli, brussels sprouts, garden peas, potatoes, and rice bran [80]. Interestingly, it was evaluated in in vitro and in vivo stroke models. All the tested dose of α-LA decreased infarct volume and brain edema and improved neurological function. The best neuroprotective effects were obtained with the α-LA dose 40 mg/kg. α-LA mitigated the MCAO induced oxidative stress, as demonstrated by the increase in SOD and GPx activities and the reduction of MDA. The anti-oxidant effects of α-LA was linked to its capacity to activate Nrf2 and HO-1. In vitro, pretreatment of α-LA reduced cytotoxicity in a dose-dependent manner and 100 μM α-LA exerted the best effect, while 1 µM was not efficacious in increasing cell viability. Accordingly, similar to the in vivo results, also in in vitro a decrease in intracellular ROS by α-LA was reported together with the increase in HO-1 and nuclear Nrf2 levels dose-dependently. Interestingly, knockdown of Nrf2 or HO-1 inhibited the neuroprotective effect of α-LA, indicating the necessity of the activation of Nrf2 signaling [81].

Isoquercetin (Iso) (C_21_H_20_O_12_; CAS number 482-35-9; Figure 6 (30)) is present in various medicinal and dietary plants, including fruits and vegetables and derived drinks [82]. Dai et al. evaluated the mechanisms of the protection exerted by Iso in I/R injured rats. Rats subjected to MCAO administered with Iso showed a decrease of infarct size and brain edema, especially in rats receiving the highest tested dose, that for this reason was used in the other experiments. Iso also ameliorated neurological score. Iso treatment exerted an antioxidant action, decreasing ROS and MDA levels and increasing SOD and CAT levels in the hippocampi of MCAO rats. The treatment with Iso decreased apoptosis and induced the translocation of Nrf2 into the nucleus, while inhibiting NOX4/ROS/NF-κB pathway. The same result was obtained also in vitro in hippocampal neurons exposed to OGD. Interestingly, Nrf2 knockdown suppressed Iso protective effects [83].

Corilagin (CL) (C_27_H_22_O_18_; CAS number 23094-69-1; Figure 6 (31)) is a phytochemical found in *Emblica officinalis Gaertn*. or *Phyllanthus emblica Linn*, better known with the name of Indian gooseberry or amla. It was used as a medicinal plant, but the fruit is also used for cooking to make vegetable dishes and fresh juice [84]. Post-treatment with CL reduced infarct volume and apoptosis and improved neurologic score. CL treatment induced also angiogenesis and increased the vascular endothelial growth factor (VEGF) and VEGFR2 expression. CL exerted also an antioxidant action, showed by the reduction of MDA levels and the increase of SOD and GPx activities, together with nuclear Nrf2 and HO-1 levels. Interestingly, the protective actions exerted by CL were Nrf2 dependent, indeed Nrf2 silencing suppressed CL protection. Also, in in vitro neurons were exposed to OGD, CL increased cell viability in a dose-dependent manner and increased also Nrf2 levels, but these effects were blocked by Nrf2 knockdown [85].

6″-*O*-succinylapigenin (C_25_H_23_O_13_; Figure 6 (32)) derived from apigenin is present in different herbs. Zhang et al. found that 6″-*O*-succinylapigenin administration decreased infarct volume and neurological deficits in MCAO rats. Moreover, it exerts antioxidant actions as demonstrated by the increase in SOD and HO-1 levels and the decrease of MDA. In addition, 6″-*O*-succinylapigenin restored phosphorylated ERK/ERK levels. However, the protein levels of total Nrf2 were not modified in vivo, even if Keap1 decreased. In vitro, both 6″-*O*-succinylapigenin and apigenin increased cell viability after OGD. However, in vitro nuclear Nrf2 increased, together with HO-1 and phosphorylated ERK/ERK, while the total Nrf2 level was not changed [86].

Fucoxanthin (C_42_H_58_O_6_; CAS number 3351-86-8; Figure 6 (33)) belongs to marine carotenoids and it is present in edible brown seaweeds. Pretreatment with fucoxanthin of rats subjected to MCAO ameliorated neurological deficits while decreased infarct volume, cerebral edema, and apoptosis in brain tissues in a dose-dependent manner. Fucoxanthin also exerted antioxidant effects, reducing ROS and MDA levels and increasing SOD activity. Fucoxanthin exerted protective effects in neurons exposed to OGD, decreasing apoptosis and oxidative stress activating Nrf2/HO-1 pathway, as demonstrated also in vivo. Indeed, Nrf2 knockdown suppressed the positive actions of fucoxanthin in neurons exposed to OGD [87].

Artichoke (*Cynara scolymus*) also is rich in compounds that showed health promoting effects and luteoloside (C_21_H_20_O_11_; CAS number 5373-11-5; Figure 6 (34)), also known as cynaroside is one of the major flavonoids [88], but it is present also in other plants. Luteoloside ameliorated neurological score, infarct volume, cerebral edema, and morphological changes in rats subjected to MCAO. The protective effects of luteoloside were induced by the inhibition of neuroinflammation, as demonstrated by reduced pro-inflammatory markers IL-1β, TNF-α, inducible nitric oxide synthase (iNOS), and COX-2 brain levels as a consequence of NF-κB signaling suppression in MCAO rats. Moreover, luteoloside increased peroxisome proliferator activated receptor gamma (PPARγ) and Nrf2 translocation into the nucleus in animals subjected to MCAO [89].

Similarly, monascin (C_21_H_26_O_5_; CAS number 21516-68-7; Figure 6 (35)), a component of red yeast rice, showed neuroprotection in an ICH model through the increase of PPARγ and Nrf2 levels. Moreover, high dosage of monascin improved neurological function, reduced BBB permeability, edema, and the volume of hematoma [90].

### 3.6. Ginseng Bioactive Compounds

Ginseng (*Panax ginseng*) is a well-known plant used as medicinal and nutritional supplements, where the main bioactive components are ginsenosides. It was used for different disorders, including stroke [91]. Korean red ginseng derived from *Panax ginseng*. Ginseng pretreatment ameliorated sensorimotor deficits during the acute stage of ischemic stroke, but also improved long-term functional rescue. Ginseng treatment was also able to reduce infarct volume on day 3. The positive effects of ginseng at least partially involved Nrf2. Indeed, ginseng pre-treatment increased the levels of HO-1, SOD2, NQO1, and GPx1. However, ginseng did not exert its positive effects in Nrf2^−/−^ mice, that showed a further exacerbated ischemic condition in comparison with WT controls. Ginseng pre-treatment was also able to attenuate reactive astrogliosis, but not microglia activation, and astrocytic dysfunctions in glutamate metabolism and water homeostasis in WT mice. The results then suggested that ginseng pretreatment may exert protection after permanent distal MCAO (pdMCAO) and Nrf2 pathway had a role in its protective effects [92]. The effects of Korean red ginseng were also evaluated 28 days after the induction of pdMCAO model. Also in this case, ginseng exerted its action on the infarct volume, but also on the reactive microgliosis and astrogliosis in the peri-infarct cortex. Also aquaporin 4 (AQP4) levels, through which astrocytes maintain brain–water homeostasis, were reduced in the ginseng pre-administered ischemic WT mice, but not in the Nrf2^−/−^ group. Then these results evidenced the importance of Nrf2 in the long-term recovery and in ginseng neuroprotective effects [93]. Recently, Liu et al. also evaluated the effects of ginseng, but also dimethyl fumarate (DMF), in a cerebral hypoxia-ischemia model and evaluated the involvement of Nrf2. Both Ginseng or DMF reduced neurological deficits, brain edema, and infarct volume in WT mice, while they did not have effects on Nrf2^−/−^ mice. On the contrary Nrf2^−/−^ animals showed a more severe condition. Both pre-treatments increased also the cortex and striatum levels of different Nrf2 downstream proteins, including NQO1, HO-1, GPx1, and SOD2 in comparison with WT controls. On the contrary, these effects were not evident in Nrf2^−/−^ mice after ischemia. Ginseng or DMF decreased the cortex and striatum levels of several pro-inflammatory mediators, including iNOS and IL-1β while the levels of the anti-inflammatory cytokine IL-10 were augmented in WT mice. In line with the reduction of pro-inflammatory mediators, also the reactive gliosis, astrocytic dysfunction in glutamate metabolism and in water homeostasis were reduced by Ginseng or DMF treatment in WT mice, but not in Nrf2^−/−^ mice. These effects were evident both 6 h and 24 h after the ischemic insult, but the neuroprotective properties were also evident in a later phase until 7 days [94]. The effects of ginseng and DMF were evaluated also in the hippocampus. DMF or ginseng pretreatment was able to reduce infarct volume as well as brain edema and hippocampal CA1 neuronal degeneration 24 h after injury in WT but not in Nrf2^−/−^ mice. Moreover, NQO1, HO-1, GPx1, and SOD2 levels increased in WT, but did not change in Nrf2^−/−^ mice. The ginseng neuroprotection was exerted both at 6 h and a week after the insult. In particular, in both early and late stages, ginseng and DMF pretreatments attenuated reactive gliosis and deficits in glutamate metabolism and regulation of water transport in WT, but not Nrf2^−/−^ mice [95].

**Figure 7:**
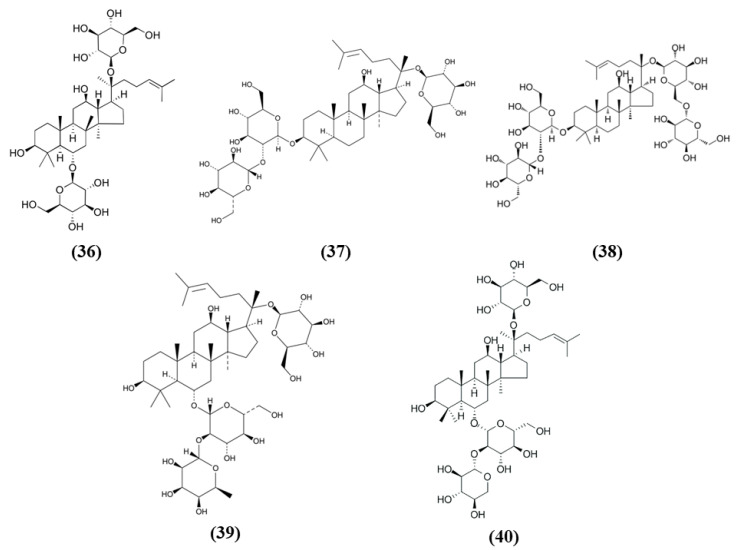
Chemical structure of (**36**) ginsenoside Rg1, (**37**) ginsenoside Rd, (**38**) ginsenoside Rb1, (**39**) ginsenoside Re, and (**40**) notoginsenoside R1.

Ginsenoside Rg1 (Rg1) (C_42_H_72_O_14_; CAS number 22427-39-0; Figure 7 (36)) is also obtained from *Panax ginseng* and belongs to the saponins. Administration of Rg1 alleviated ischemic damage, decreasing infarct volume and reducing oxidative stress. Rg1 activated Nrf2 pathway in MCAO rats, increasing nuclear Nrf2 and NQO1, HO-1, GCLC, and GCLM. Similar results were obtained when miR-144 was inhibited. In PC12 cells exposed to OGD, Rg1 exerted protective effects improving cell viability and decreasing ROS levels. In addition, in vitro Rg1 prolonged Nrf2 translocation to the nucleus, increasing the expression of its downstream targets. Interestingly, Nrf2 silencing suppressed the protection exerted by Rg1, indicating that the protective effects of Rg1 depend on Nrf2. However, Keap1 seemed not to be involved in the modulation of Nrf2 by Rg1. Instead, OGD caused an increase in miR-144, on the contrary Rg1 administration reduced it. In particular, miR-144 targeted 3′-untranslated region of Nrf2 causing its downregulation after OGD. All together, these results indicated that Rg1 ameliorated oxidative stress in these experimental models through the inhibition of miR-144 and as a consequence inducing Nrf2 signaling [96].

Xueshuantong injection (lyophilized, XST) is derived from *Panax notoginseng* and its principal compounds are panaxadiol saponin and panaxatriol saponins, including ginsenoside Rd (C_48_H_82_O_18_; CAS number 52705-93-8; Figure 7 (37)) and ginsenoside Rb1 (C_54_H_92_O_23_; CAS number 41753-43-9; Figure 7 (38)), and Rg1, ginsenoside Re (C_48_H_82_O_18_; CAS number 52286-59-6; Figure 7 (39)) and notoginsenoside R1 (C_47_H_80_O_18_; CAS number 80418-24-2; Figure 7 (40)), respectively. XST improved neuronal functional deficit time- and dose-dependently, indicating that longer administration may exert better improvements. The administration of 100 mg/kg XST increased vascular density and angiogenesis as demonstrated by the increase of VEGF as well as fibroblast growth factor (FGF)-2. In addition, XST showed antioxidant actions, increasing CAT activity and total antioxidant capacity. XST protection was due to the increase of Nrf2, HO-1, and NQO1 expressions, indicating a role of Nrf2 pathway [97].

### 3.7. Salvia Miltiorrhiza Bioactive Compounds

**Figure 8:**
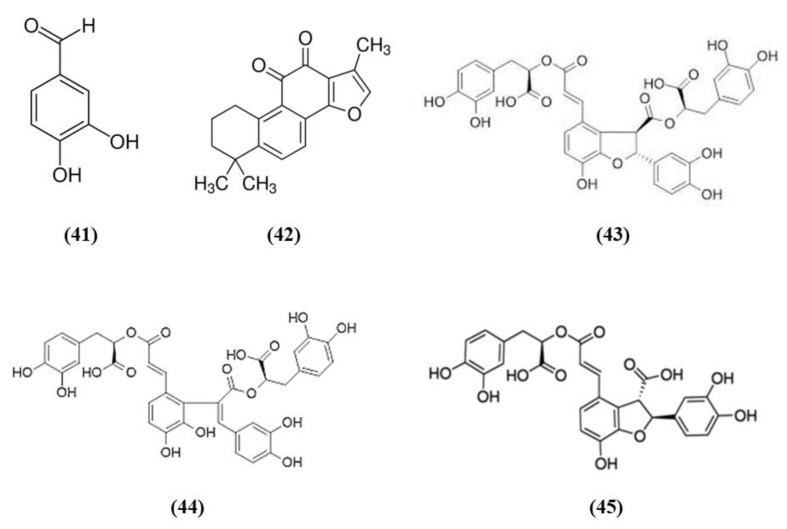
Chemical structure of (**41**) protocatechualdehyde, (**42**) tanshinone IIA, (**43**) salvianolic acid B, (**44**) salvianolic acid E, and (**45**) lithospermic acid.

Salvia species contain different compounds with beneficial effects and are widely used in food and pharmaceutical products [98]. In particular, *Salvia miltiorrhiza*, also known as Danshen, is a popular medicinal plant used also for the treatment of cerebrovascular diseases and its bioactive compounds exert health promoting effects [99]. Among the bioactive compounds of *Salvia miltiorrhiza*, protocatechualdehyde (PCA) (C_7_H_6_O_3_; CAS number 139-85-5; Figure 8 (41)), and tanshinone IIA (TSA) (C_19_H_18_O_3_; CAS number 568-72-9; Figure 8 (42)) were tested in stroke animal models.

Guo et al. showed that PCA was able to counteract oxidative stress in a cerebral I/R model through the PKCε/Nrf2/HO-1 pathway. Specifically, the PCA administration induced an improvement of neurological deficits, associated with a reduction of infarct volume and necrosis. PCA exerted an antioxidant action, demonstrated by the reduction of ROS, 4-HNE, and 8-OHdG levels. In parallel, PCA upregulated the nuclear levels of Nrf2 and also HO-1 in cortical neurons. PCA protective effects were also observed in differentiated SH-SY5Y cells exposed to OGD. Indeed, PCA was able to increase cell viability and the optimal concentration was 80 μM. PCA exposure increased Nrf2 and HO-1 levels, in the nucleus and cytoplasm, respectively, in a dose-dependent manner. Interestingly, the neuroprotection exerted by PCA was suppressed by Nrf2 or HO-1 knocking down, indicating that the protective effects of PCA depend on Nrf2. Interestingly, PCA protective effects involved PKCε increase, indeed its knockdown suppressed Nfr2 translocation into the nucleus, HO-1 expression, and consequently PCA neuroprotection [100].

Similarly, also TSA administration exerted protective effects acting on neurological deficits, infarct volume and neuronal apoptosis. The protection is mediated by antioxidant actions, showed by the reduction in the levels of carbonyl protein, nitrotyrosine, 8-OHdG as well as MDA. On the contrary, antioxidant enzyme levels SOD, CAT, and GPx and total-antioxidant capacity were increased. In association, TSA treatment up-regulated Nrf2 expression and translocation into the nucleus. Interestingly, both Nrf2 knockdown or Nrf2 knockout suppressed TSA antioxidant and protective actions, demonstrating that they depend on Nrf2 pathway [101].

Salvianolate lyophilized injection (SLI) is obtained from *Salvia miltiorrhiza* aqueous extract, composed mainly of salvianolic acid B (C_36_H_30_O_16_; CAS number 121521-90-2; Figure 8 (43)), salvianolic acid E (C_36_H_30_O_16_; CAS number 142998-46-7; Figure 8 (44)), lithospermic acid (C_27_H_22_O_12_; CAS number 28831-65-4; Figure 8 (45)), and RA. SLI was able to induce protective effects in a stroke model in type 1 diabetic rats. Diabetes mellitus is known to increase the risk for ischemic stroke, leading to bad outcomes and increasing mortality. SLI treatment reduced neuronal damage, increased brain microvasculature and glucose uptake in different areas of the brain. SLI also significantly decreased RAGE, MMP9 levels, as well as the levels of the pro-inflammatory mediators COX-2, TNF-α, and intercellular adhesion molecule 1 (ICAM-1). The authors also evidenced that Nrf-2 pathway was implicated in SLI protective effects, indeed an increase in HO-1, NAD(P)H quinine oxidoreductase (HQO-1), and Nrf2 was observed in MCAO rats treated with SLI [102].

### 3.8. Ginkgo biloba Bioactive Compounds

**Figure 9:**
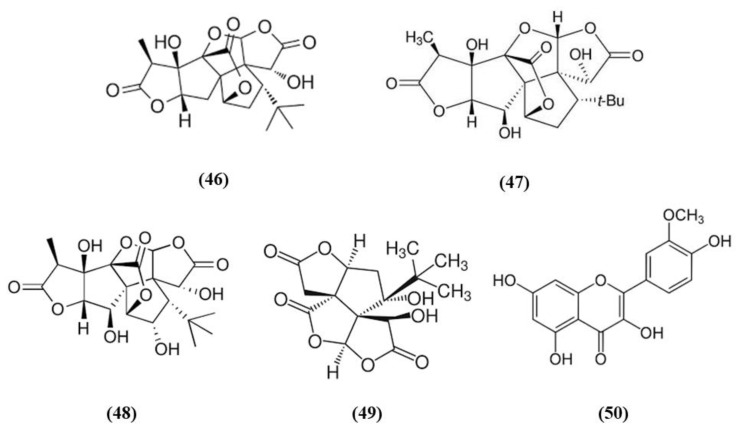
Chemical structure of (**46**) ginkgolides A, (**47**) ginkgolides B, (**48**) ginkgolides C, (**49**) bilobalide, and (**50**) isorhamnetin.

*Ginkgo biloba* is a widely used dietary supplement also for vascular protection [103]. Diterpene ginkgolides meglumine injection (DGMI) is obtained from *Ginkgo biloba* L., and is composed by ginkgolides A (GA) (C_20_H_24_O_9_; CAS number 15291-75-5; Figure 9 (46)), B (GB) (C_20_H_24_O_10_; CAS number 15291-77-7; Figure 9 (47)) and C (GC) (C_20_H_24_O_11_; CAS number 15291-76-6; Figure 9 (48)). DGMI was able to improve dose-dependently the neurological function, but also decreased infarct volume and cerebral edema when administered at medium and high concentrations. Also apoptosis induced by ischemic stroke was attenuated by DGMI treatment. Both concentrations were also able to increase Akt phosphorylation, Nrf2 levels in the nucleus, and its target HO-1. In parallel, DGMI treatment increased also the levels of phosphorylation of the survival-modulating protein cyclic AMP-responsive element binding protein (CREB). In addition, treatment with DGMI or GA, GB or GC significantly reduced cell death in PC12 cells exposed to OGD. Also in vitro treatment with DGMI or with GA, GB, or GC induced an increase in the levels of phosphorylated Akt, Nrf2 in the nucleus and activated CREB, but these effects were reversed by a PI3K inhibitor. These findings suggested that ginkgolides activate the Nrf2 pathway in a PI3K/Akt-dependent manner to exert protection in I/R damage experimental models [104].

In addition to the previous study, GB and bilobalide (BB) (C_15_H_18_O_8_; CAS number 33570-04-6; Figure 9 (49)) were shown to decrease ROS levels, but only GB increased SOD activity in OGD-exposed SH-SY5Y. GB, GK, and BB induced an increase in HO-1 expression, while GB, GA, and BB increased NQO1 expression. GA, GB, and BB were also able to increase the levels of phosphorylated Akt and Nrf2. Interestingly, GB treatment exerted advantages compared to the other compounds for all the parameters. However, a PI3K inhibitor reversed GB effects on phosphorylated Akt, phosphorylated Nrf2, and cell viability. This result indicated that the beneficial actions depend on Akt/Nrf2 pathway. In vivo, treatment with GB decreased infarct volume dose-dependently in MCAO rats. GB treatment increased the protein expression of HO-1, NQO1, SOD, phosphorylated Akt, phosphorylated and total Nrf2 in a dose-dependent manner [105].

Isorhamnetin (C_16_H_12_O_7_; CAS number 480-19-3; Figure 9 (50)) is a flavonol aglycone present in different plants including *Ginkgo biloba*. Isorhamnetin administration to mice subjected to experimental stroke decreased infarct volume, brain edema, and apoptosis as well as improved sensorimotor function. It also ameliorated BBB disruption, increasing the gene expression of occludin, ZO-1, and claudin-5. Isorhamnetin administration induced the activation of Nrf2/HO-1 signaling, promoting the translocation of Nrf2 into the nucleus and increasing HO-1 activity. Nrf2 activation was associated with a decrease of iNOS, MDA, and 3-nitrotyrosine in ipsilateral cortex. Isorhamnetin exerted also an anti-inflammatory action, abolishing the activity of myeloperoxidase evaluated as a marker of neutrophil infiltration, and reducing the levels of the proinflammatory cytokines IL-1β, IL-6, and TNF-α. In addition, considering that N-methyl-D-aspartate receptor (NMDAR), is activated in ischemic stroke causing apoptosis while its inhibition was associated to neuroprotection, the expression of the subunit NR1 was evaluated. Interestingly, isorhamnetin reduced gene expression, but also protein levels of the NR1 subunit in the ipsilateral cortex [106].

### 3.9. Bioactive Compounds of Chuanxiong Rhizome

**Figure 10:**
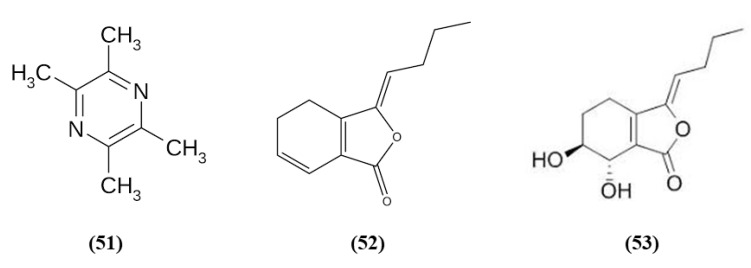
Chemical structure of (**51**) tetramethylpyrazine, (**52**) Z-ligustilide, and (**53**) senkyunolide I.

Chuanxiong Rhizome is the dried rhizome of *Ligusticum chuanxiong Hort*; it is widely used as a medicinal plant, and also as a food with health promoting properties in China [107]. Tetramethylpyrazine (TMP) (C_8_H_12_N_2_; CAS number 1124-11-4; Figure 10 (51)) is one of its active ingredients. TMP was able to inhibit inflammatory cell intracerebral infiltration and reduced neuronal loss in an experimental model of permanent cerebral ischemia. TMP also decreased ischemia-induced activation of circulating neutrophils and upregulated Nrf2 and HO-1 in neutrophils [108]. Z-ligustilide (C_12_H_14_O_2_; CAS number 81944-09-4; Figure 10 (52)) is one of the main compounds found in the volatile oil of *Rhizoma Chuanxiong*. Li et al. studied the prophylactic effects of intranasal administration of Z-ligustilide. The pre-treatment with Z-ligustilide reduced infarct volume, BBB damage, and brain edema and improved neurological function. According to the reduction of the BBB disruption, Z-ligustilide pre-treatment decreased MMP 2 and MPP 9 levels, that cause extracellular matrix degradation causing then BBB disruption and increased occludin and ZO-1. Moreover, Z-ligustilide prevented the reduction of collagen IV, present in the vascular basement membrane. Z-ligustilide also upregulated NQO1 and HSP70. However, inhibition of Nrf2 or HSP70 reduced the preventive potential of Z-ligustilide, abolishing its protective actions [109].

Senkyunolide I (SEI) (C_12_H_16_O_4_; CAS number 94596-28-8; Figure 10 (53)), also obtained from *Ligusticum chuanxiong*, represent a metabolite of Z-ligustilide, and its neuroprotective effects were evaluated in rats subjected to MCAO. The high dose of SEI improved neurological function, reduced brain edema, infarct volume, morphological abnormalities in the cortex and hippocampus, but also MDA levels while SOD activity increased. Moreover, the authors showed that the SEI high dose may induce Nrf2 activation through the up-regulation of the phosphorylated ERK1/2, that induced Nrf2 nuclear translocation, increasing as a consequence HO-1 and NQO1 levels. SEI administration showed antiapoptotic effects. It is important to notice that the lowest SEI dose was able to exert only a partial protection [110].

### 3.10. Gastrodia elata Blume Bioactive Compounds

**Figure 11:**
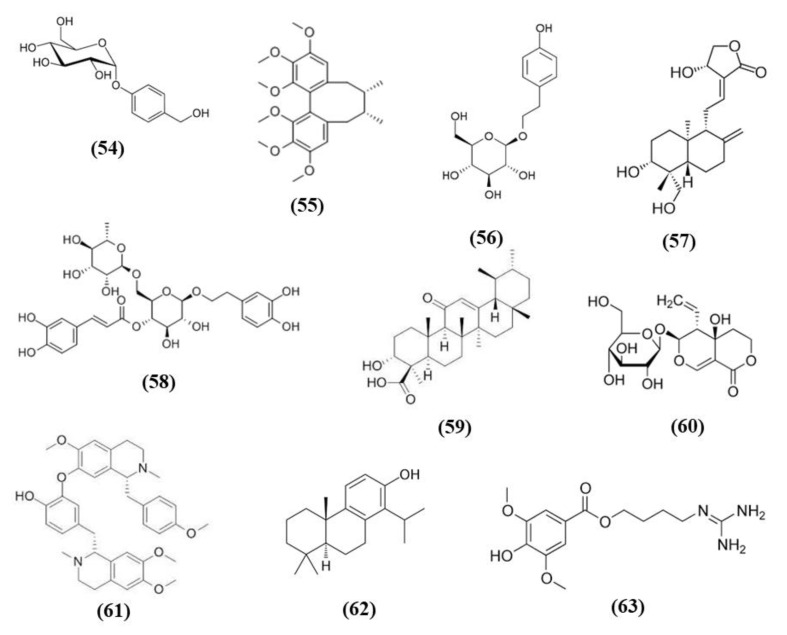
Chemical structure of (**54**) gastrodin, (**55**) schizandrin A, (**56**) salidroside, (**57**) andrographolide, (**58**) forsythiaside A, (**59**) 11-Keto-β-boswellic acid, (**60**) swertiamarin, (**61**) neferine, (**62**) totarol, and (**63**) leonurine.

*Gastrodia elata Blume* a plant of the orchidaceae family, known in China as Tian ma, is used as herbal medicine in traditional Chinese medicine, given that its bioactive compounds exerted different health promoting effects, but it is used also a functional food [111]. Gastrodin (GAS) (C_13_H_18_O_7_; CAS number 62499-27-8; Figure 11 (54)) represents its principal phenolic compound obtained from the root. GAS decreased infarct volume, apoptosis, and neurobehavioral deficit in MCAO mice, in particular when administered at high dose. These effects were evident 24 h after reperfusion, but they were long-lasting. Indeed, these protective actions were also present 7 days after reperfusion, in mice that received GAS every day. Moreover, GAS treatment exerted antioxidant and anti-inflammatory effects as evidenced by the decrease of MDA, of pro-inflammatory cytokines, and by the increase of SOD activity and of HO-1 and SOD1 levels in the brain of mice receiving GAS. In addition, GAS increased the levels of phosphorylated Akt and Nrf2 [112].

Another study demonstrated that GAS neuroprotection was associated with the protection from the toxicity of Zn^2+^ and to the antioxidant action in astrocytes. GAS treatment after MCAO decreased infarct volume and ameliorated neurological function when administered after 1 h or after 6 h, indicating that GAS was able to counteract ischemic damage in a wide therapeutic window. The dose 40 mg/kg exerted the best neuroprotective effects. Gas also exhibited neuroprotective effects in vitro, increasing cell viability of C6 astrocytic cells exposed to Zn^2+^, given that Zn^2+^ was reported to exert toxicity during stroke, even if in normal conditions it is an important cofactor of different enzymes. In association, GAS up-regulated Nrf2 in the nucleus, and as a consequence increased the expression of its downstream targets, including HO-1 and GCLM in astrocytes. Accordingly, GAS reduced also ROS production, Poly [ADP-ribose] polymerase 1 (PARP-1) induction, and p67 expression. These data suggested that GAS exerted antioxidant effects that may induce protection in the ischemic brain [113].

The pre-treatment with the phenolic components of *Gastrodia elata Blume* (PCGE) reduced motor impairment and ameliorated cognitive function and pathological lesions in rats subjected to MCAO. In addition, the amounts of MAP2 positive dendrites and of surviving neurons and astrocytes increased. PCGE reduced also H_2_O_2_ toxicity in astrocytes and SH-SY5Y cells, showing antioxidant effects increasing the levels of nuclear Nrf2, HO-1, and NQO1. Also an increase in BDNF, a neurotrophic factor that depends on Nrf2, was found in PCGE-treated astrocytes [114].

GAS administration was also evaluated in a rat ICH model. GAS improved brain edema at 72 h after ICH and improved neurological function after 24 and 72 h. Moreover, GAS administration counteracted oxidative stress reducing ROS, 8-OHdG, 3-nitrotyrosine, and MDA levels, and increasing GPx and SOD activities. In line with the antioxidant action, GAS upregulated Keap1/Nrf2/HO-1 after 72 h. At the same time, GAS exerted an anti-apoptotic action in the perihematoma area. Also in vitro GAS exerted protective effects, being able to reduce the apoptosis caused by hematoma lysate in cortical neurons [115].

### 3.11. Other Plant-Derived Bioactive Compounds

*Schisandra chinensis* is a plant whose fruits are used as traditional Chinese medicine. It is rich in different bioactive compounds known for the positive effects. However, it is also used in food technology as additives to enhance the flavor and taste as well as the nutritional value of food [116]. Schizandrin A (Sch A) (C_24_H_32_O_6_; CAS number 61281-38-7; Figure 11 (55)), obtained from *Schisandra chinensis,* was shown to have anti-inflammatory and antioxidant activities in vivo and in vitro models. Sch A treatment ameliorated neurological function and decreased infarct volume, with the highest dose that showed the best effects. Sch A inhibited COX-2 and iNOS expression, decreased the levels of the pro-inflammatory cytokines TNF-α, IL-1β, and IL-6, while increasing the levels of TGF-β and IL-10 that exert an anti-inflammatory action. SOD and CAT activities were increased, while ROS, 4-HNE, and 8-OHdG levels were reduced. The antioxidant effects were due to the increase of Nrf2 in the nucleus, together with its targets HO-1 and NQO-1. SchA also decreased the loss of cell viability in vitro in a OGD model, together with pro-inflammatory markers, while Nrf2, HO-1, and NQO1 expression increased. Interestingly, knock¬down of Nrf2 suppressed Sch A neuroprotection. Sch A also improved adenosine monophosphate-activated protein kinase (AMPK) phosphorylation in both experimental models. AMPK knockdown blocked Nrf2 activation, indicating that Sch A protective effects involved AMPK/Nrf2 pathway [117].

*Rhodiola* plants possess both edible and medicinal value and are considered functional food [118]. Salidroside (C_14_H_20_O_7_; CAS number 10338-51-9; Figure 11 (56)) is a constituent of *Rhodiola*. Salidroside exerted neuroprotection in a MCAO model. Indeed, the highest dose of salidroside reduced neurological score and infarct volume while ameliorating the morphology of neuronal cells in cortex and striatum. The protective effects were due to the increase of Nrf2 and HO-1. In addition, the activities of SOD and GST increased, while MDA levels decreased [119]. Salidroside actions were also tested in a pMCAO model. Salidroside administration in pMCAO rats reduced infarct volumes and neurological deficits. It is important to notice that only the highest dose tested was able to induce these protective effects and for this reason the further analyses were performed using only this dose. Salidroside increased nuclear Nrf2, and its target gene HO-1. In parallel, it decreased NF-κB subunit p50 nuclear levels and the gene expression of IL-6 as well as TNFα. Nrf2 inhibition suppressed the protective action of salidroside. Salidroside exerted its effects also increasing p-PKB/PKB ratio and the use of a PI3K inhibitor inhibited all the protective actions of salidroside. Then the results indicated that salidroside decreased neuronal damage through the modulation of PI3K/PKB/Nrf2/NFκB pathway [120].

Wang et al. showed that total glycosides (TGs) of *Cistanche deserticola* exerted protective effects in ischemic stroke. Indeed, TGs treatment decreased neurological deficits and infarct volumes and improved histological damage. Interestingly TGs were also able to ameliorate BBB integrity, increasing claudin 5, occludin, and ZO-1 expressions and pericyte coverage on capillaries. TGs also promoted angiogenesis and neural remodeling. The protective effects of TGs were associated with its antioxidant effects. Indeed, TGs administration reduced MDA levels, while the cerebral SOD, CAT, and GPx activities were increased in association with the increase of nuclear Nrf2 and the reduction of Keap1. The polysaccharides and oligosaccharides extracts of *Cistanche deserticola* were not able to exert neuroprotective effects [121].

Andrographolide (C_20_H_30_O_5_; CAS number 5508-58-7; Figure 11 (57)) represents the major active compound of *Andrographis paniculata* leaf extracts and Yen et al. evaluated its effects in cerebral endothelial cells (CECs) and in rats subjected to MCAO. In vitro, andrographolide was able to increase HO-1, phosphorylated and nuclear Nrf2 levels in CECs, through the mediation of p38, while ERK or JNK were not involved. Andrographolide reduced cell death induced by OGD in CECs through HO-1 signaling, indeed its inhibition abolished the protective effects. In vivo, andrographolide reduced free radical formation, BBB disruption, and infarct volume, but these protective effects were inhibited in the presence of HO-1 inhibitor, indicating that HO-1 is needed for andrographolide protective effects. These findings suggested that andrographolide increased Nrf2/HO-1 signaling via p38 exerting protection in MCAO rats [122].

Forsythiaside A (FA) (C_29_H_36_O_15_; CAS number 79916-77-1; Figure 11 (58)) is one of the main compound present in the fruits of *Forsythia suspensa* and it was able to exert protective actions in a rat MCAO model. FA increased the survival rate while decreased neurological deficits as well as apoptosis. FA showed antioxidant actions increasing the protein levels of Nrf2, NQO1, and GST. Moreover, FA reduced serum MDA and increased SOD and GSH. In addition, FA reduced endoplasmic reticulum stress [123].

11-Keto-β-boswellic acid (KBA) (C_30_H_46_O_4_; CAS number 17019-92-0; Figure 11 (59)) is a triterpen extracts from *Boswellia serrata*. KBA administration decreased infarct volume, apoptosis, and ameliorated neurologic score. KBA exerted antioxidant function, as shown by reduced MDA levels and increased SOD and GPx activities, in parallel to the increased Nrf2 and HO-1. In primary astrocytes, KBA also augmented nuclear Nrf2 and HO-1 levels and exerted protective and antioxidant effects against OGD. Interestingly, Nrf2 or HO-1 knockdown inhibited KBA protection, indicating their involvement in KBA beneficial effects [124].

Swertiamarin (Swe) (C_16_H_22_O_10_; CAS number 17388-39-5; Figure 11 (60)) is isolated from *Gentiana macrophylla Pall*. Swe pre-treatment reduced infarct volume, apoptosis, and oxidative stress in association with an improvement in neurologic function. The antioxidant action was also demonstrated by the increase of SOD, CAT, and GPx activities and by the reduction of MDA levels. In addition, NQO1 and HO-1 and the nuclear expression of Nrf2 were also increased, while Keap1 levels decreased. Also in vitro Swe was able to decrease oxidative stress. Interestingly, a Nrf2 inhibitor suppressed the protective effects of Swe in vitro. It was suggested that Swe caused the release of Nrf2 from its complex with Keap1 [125].

Neferine (Nef) (C_38_H_44_N_2_O_6_; CAS number 2292-16-2; Figure 11 (61)) is derived from *Nelumbo nucifera Gaertn* seeds. In vitro, Nef was able to protect PC12 cells from tert-butyl hydroperoxide cytotoxicity. In vivo, Nef treatment in rats subjected to pMCAO improved neurological function, infarct volume, regional cerebral blood flow, and cerebral microstructure. Moreover, Nef exerted an antioxidant action preventing mitochondrial damage in both experimental models. In particular, it mediated the mitochondrial protection through the increase of nuclear Nrf2, HO-1, and NQO1. Blocking Nrf2 signaling abolished Nef protective effects. Interestingly, Nef induced also autophagy and an association between Nrf2 and autophagy was suggested. In particular, the pathway p62/Keap1/Nrf2 seemed to have a role in Nrf2 activation [126].

Totarol (C_20_H_30_O; CAS number 511-15-9; Figure 11 (62)) is a phenolic diterpenoid isolated from *Podocarpus totara*. Gao et al. reported that totarol prevented glutamate and OGD induced loss of cell viability in primary rat cerebellar granule neuronal cells and cortical neurons. Totarol activated Akt/GSK-3β pathways and increased Nrf2 and HO-1 levels, suppressing oxidative stress also through the increase of GSH and of SOD activity. The use of PI3K/Akt and HO-1 inhibitors suppressed totarol neuroprotection. In vivo, in rats subjected to MCAO model, totarol decreased infarct volume and ameliorated neurological function. Also in vivo totarol upregulated HO-1 expression and the levels of GSH and SOD activity [127].

Leonurine (C_14_H_21_N_3_O_5_; CAS number 24697-74-3; Figure 11 (63)) is a bioactive component of *Herba leonuri*. Xie et al. evidenced that leonurine (10 mg/kg) decreased infarct volume and ameliorated neurological function in a pMCAO model. The effects of leonurine were associated with Nrf2 pathway. Indeed, both total and nuclear Nrf2 increased, together with SOD, CAT, GSH, and GPx, while MDA and ROS levels decreased. In addition, leonurine upregulated VEGF expression in different cell types including neurons, astrocytes, and endothelial cells. Notably, leonurine showed no protection in Nrf-2^−/−^ mice, indicating that its beneficial effects depend on Nrf2 signaling [128].

**Figure 12:**
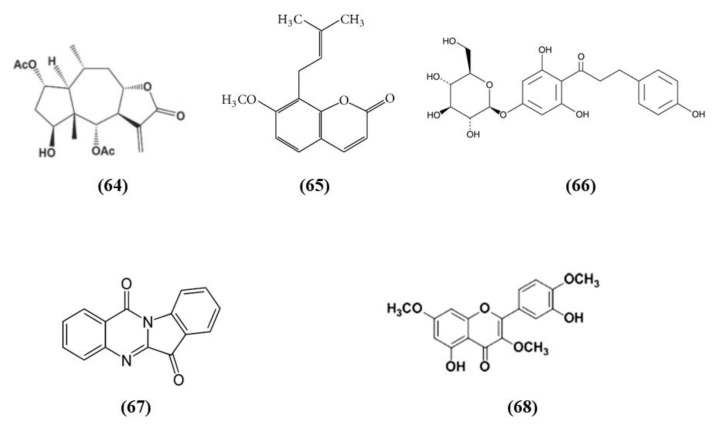
Chemical structure of (**64**) britanin, (**65**) osthole, (**66**) trilobatin, (**67**) tryptanthrin, and (**68**) 5,3′-dihydroxy-3,7,4′-trimethoxyflavone.

Wu et al. found that britanin (C_19_H_26_O_7_; CAS number 33627-28-0; Figure 12 (64)), originally isolated from *Inula lineariifolia*, represents a strong modulator of Nrf2. In vitro, in cortical neurons subjected to OGD, pre- or post-treatment with britanin induced protective effects. In particular, the britanin-induced protection depends on Nrf2 activation. Britanin increased both Nrf2 and NQO1 levels. But also in vivo britanin ameliorated MCAO injury, reducing infarct volume, improving neurological deficits, and increasing Nrf2, NQO1, and HO-1 levels. Unfortunately, britanin showed a narrow therapeutic window. Indeed, britanin administrated 4 h after reperfusion did not exert protection. In particular, the authors evidenced that britanin binds Keap1, inhibiting Keap1-mediated ubiquitination of Nrf2, inducing in this way the Nrf2 pathway [129].

Osthole (C_15_H_16_O_3_; CAS number 484-12-8; Figure 12 (65)), a major compound obtained from *Cnidium monnieri* (L.) *Cusson*, improved cognitive function and ameliorated BBB disruption and hippocampus histological changes caused by cerebral ischemia. Osthole exerted also antioxidant action, increasing SOD activity and decreasing MDA levels. Indeed, in vitro it was able to activate Nrf2 [130].

Trilobatin (TLB) (C_21_H_24_O_10_; CAS number 4192-90-9; Figure 12 (66)) derived from *Lithocarpus polystachyus* and Gao et al. evidenced that it ameliorated neurological deficits, cerebral edema, infarct volume in the group of MCAO rats. Furthermore, they evaluated also the time window for TLB administration after MCAO, indicating that TLB (20 mg/kg) exerted protection after MCAO within 4 h, while, the protection decreased when it was administrated at 6 h after MCAO. Interestingly, TLB was able to restore long-term neurological functions. The neuroprotection exerted by TLB was mediated by the suppression of inflammation, evidenced by the reduced astrocyte and microglia activation and of the pro-inflammatory marker levels. TLB also reduced ROS and MDA levels while elevated SOD and GPx activities. The antioxidant action was associated with the upregulation of nuclear Nrf2, NQO1, and HO-1 expression together with the reduction of Keap1. TLB also increased Sirt3 expression. TLB was also able to exert protective antioxidant and anti-inflammatory effects in astrocytes and cortical neurons exposed to OGD in vitro [131].

Achyranthes bidentata polypeptide k (ABPPk) was isolated from *Achyranthes bidentata Bl. (A. bidentata*). Pretreatment with ABPPk was able to exert anti-inflammatory actions in BV2 microglia cells treated with LPS. In particular, iNOS and NO levels were reduced together with NF-κB and consequently pro-inflammatory cytokines. Interestingly, the levels of HO-1 and Nrf2 increased. In particular, the protective actions of ABPPk were mediated by Nrf2, indeed its knock down reduced ABPPk effects [132]. Similar results were obtained by Kwon et al. using tryptanthrin (C_15_H_8_N_2_O_2_; CAS number 13220-57-0; Figure 12 (67)) in BV2 cells exposed to LPS. In addition, tryptanthrin was able to suppress the induction of M1 phenotype in microglia. Also in this case the effects seem to be mediated at least in part by Nrf2/HO-1 [133].

5,3′-dihydroxy-3,7,4′-trimethoxyflavone (DTMF) (C_18_H_16_O_7_; Figure 12 (68)) derived from *Siegesbeckia pubescens* was tested in mouse hippocampal HT22 and microglia BV2 cells exposed respectively to glutamate and LPS. DTMF reduced glutamate cytotoxicity and oxidative stress. In BV2 cells, DTMF reduced iNOS, COX2, and pro-inflammatory cytokines. In both cell types, DTMF increased HO-1 and Nrf2 nuclear translocation [134].

Longxuetongluo capsule (LTC) derived from *Dracaena cochinchinensis* was shown to exert protective effects reducing the loss of cell viability in two different in vitro models, that are BV2 cells exposed to OGD or LPS. In parallel, LTC treatment also decreased pro-inflammatory markers, while Nrf2 levels in the nucleus increased, together with HO-1 levels in BV2 cells treated with LPS [135].

### 3.12. Studies with a Combination of Bioactive Compounds

**Figure 13:**
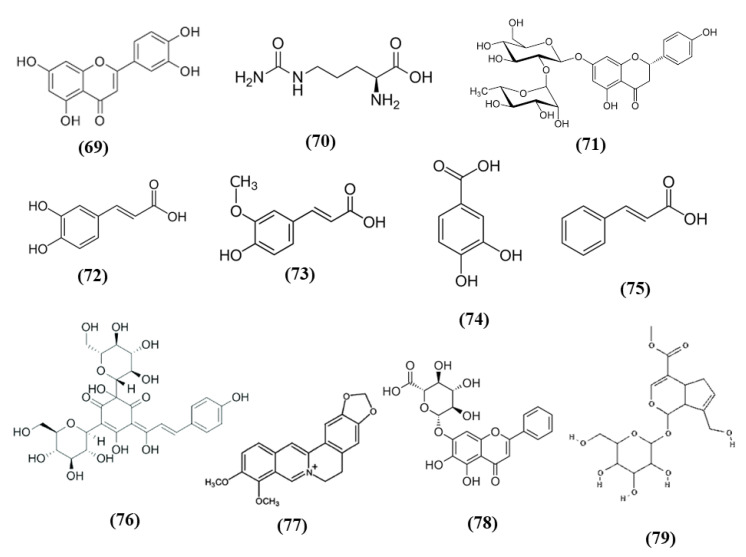
Chemical structure of (**69**) luteolin, (**70**) citrulline, (**71**) naringin, (**72**) caffeic acid, (**73**) ferulic acid, (**74**) protocatechuic acid, (**75**) cinnamic acid, (**76**) hydroxysafflor yellow A, (**77**) berberine, (**78**) baicalin, and (**79**) jasminoidin.

Gualou Guizhi granule (GLGZG) was shown to contain 104 compounds and among the bioactive components there are luteolin (C_15_H_10_O_6_; CAS number 491-70-3; Figure 13 (69)), curcumin, catechin, citrulline (C_6_H_13_N_3_O_3_; CAS number 372-75-8; Figure 13 (70)), naringin (C_27_H_32_O_14_; CAS number 10236-47-2; Figure 13 (71)), caffeic acid (C_9_H_8_O_4_; CAS number 331-39-5; Figure 13 (72)), ferulic acid (C_10_H_10_O_4_; CAS number 1135-24-6; Figure 13 (73)), protocatechuic acid (C_7_H_6_O_4_; CAS number 99-50-3; Figure 13 (74)), cinnamic acid (C_9_H_8_O_2_; CAS number 140-10-3; Figure 13 (75)). GLGZG treatment in a MCAO model reduced oxidative stress as evidenced by the increased SOD activity and levels of GSH, while ROS and MDA decreased. GLGZG induced the translocation of Nrf2 into the nucleus and increased Nrf2, HO-1, and NQO1 levels. Also in vitro GLGZG exerted antioxidant effects, activating Nrf2 signaling [136].

The protective actions of Danshensu and hydroxysafflor yellow A (HSYA) (C_27_H_32_O_16_; CAS number 78281-02-4; Figure 13 (76)) alone or in combination were evaluated in rats subjected to MCAO. Danshensu is derived from *Salvia miltiorrhiza* while HSYA is obtained from *Carthamus tinctorius* L. The combination of Danshensu and HSYA improved neurological defects, reduced infarct volume, apoptosis in cortex, and also TNF-α, IL-1β and IL-6 levels and reduced oxidative stress, as demonstrated by the reduction of the MDA level together with the increased SOD and GPx activities. Danshensu and HSYA counteracted inflammation and oxidative stress through the modulation of TLR4/NF-κB and Nrf2/HO-1 signaling. In particular, the protein nuclear levels of Nrf2 and HO-1 increased. Also in vitro in primary neurons exposed to OGD Danshensu and HSYA group exhibited neuroprotection. It is important to notice that also the single compound alone exerted protective effects, but their combination showed better neuroprotection, indicating their synergistic action [137].

Zhang et al. evaluated the beneficial action of safflower extract and aceglutamide (SAAG) in counteracting cerebral I/R damage. SAAG in combination reduced neurological deficits, infarct rate and oxidative stress. In particular, Nrf2, NQO-1, SOD, GSH, GPx, and thioredoxin levels increased after the treatment with SAAG. On the contrary MDA, nitrotyrosine and 8-OHdG levels decreased. SAAG also suppressed p38 and JNK cascades and apoptosis. The single compound also exerted some beneficial effects, but their SAAG combination was more efficacious. The SAAG exerted also protective effects in vitro in PC12 cells exposed to H_2_O_2_. Inhibition of thioredoxin and Nrf2 systems suppressed SAAG beneficial effects. The results indicated that SAAG was able to exert protective effects owing to its antioxidant effects, modulation of Nrf2, and also GSH and thioredoxin systems [138].

SLI and XST showed neuroprotective effects in stroke models. Their combination was tested in rats subjected to MCAO. Their combination increased body weights, ameliorated neurobehavioral deficits, increased regional cerebral blood flow, and reduced the lesion volumes. The protective effects were associated with the suppression of microglia and astrocyte activation in the hippocampus, but also with antioxidant effects. Indeed, the combination of SLI and XST reduced MDA and ROS levels, while the activities of SOD and CAT increased together with GSH amount in the brain tissue of MCAO rats. The antioxidant action was linked to Nrf2 increased expression and nuclear translocation, together with the increase in HO-1 and NQO1 amounts, and the downregulation of Keap1. Notably, also SLI or XST alone was able to exert protective effects, but their combination showed a stronger protection [139].

Tao Hong Si Wu decoction (THSWD) is a traditional medicine from China, composed of *Radix Rehmanniae Praeparata, Radix Angelicae Sinensis, Rhizoma Ligustici Chuanxiong, Radix Paeoniae Alba, Semen Prunus,* and *Flos Carthami Tinctorii*, which showed protective effects in I/R injury. THSWD decreased infarct volume together with neurological deficits. In parallel, it increased HO-1. THSWD was also able to increase HO-1 expression and Nrf2 translocation into the nucleus, showing that PI3K/Akt pathway participate in Nrf2 regulation [140].

Huang-Lian-Jie-Du-Decoction (HLJDD) is commonly used in traditional Chinese medicine and various combinations of the main compounds found in HLJDD, which are berberine (C_20_H_18_NO_4_^+^; CAS number 2086-83-1; Figure 13 (77)), baicalin (C_21_H_18_O_11_; CAS number 21967-41-9; Figure 13 (78)), and jasminoidin (C_17_H_24_O_10_; CAS number 169799-41-1; Figure 13 (79)) were tested for their therapeutic efficacy against ischemic stroke. The combinations of the three compounds decreased infarct volume and ameliorated neurological score. Moreover, the combination upregulated the levels of cellular antioxidants, including SOD, GSH, GPx, and reduced GSSG and MDA levels, through the activation of Nrf2. Indeed, Nrf2 and HO-1 expression increased, while Keap1 expression decreased. In line with the protective effects of the combination, markers of inflammation and apoptosis decreased. It is important to notice that also each compound alone, or combinations with only two of them, were able to exert a partial protection, but the combination of berberine, baicalin, and jasminoidin together showed the strongest protective effects [141].

**Figure 14:**
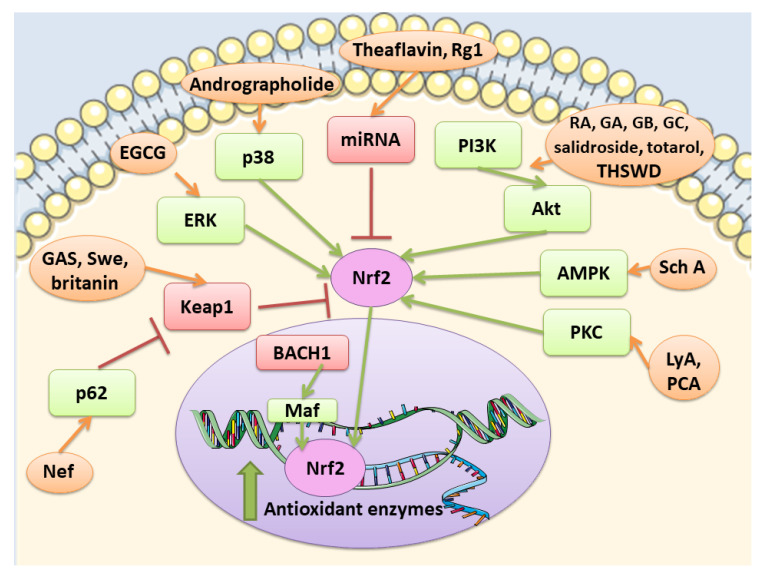
Schematic representation of the different pathways activated by the natural compounds that are involved in Nrf2 modulation. The orange ovals indicate the compounds that activated each pathway. The figure was made taking the images from Servier Medical Art (available at http://smart.servier.com/), licensed under a Creative Commons Attribution 3.0 Unported License (https://creativecommons.org/licenses/by/3.0/). AMPK, monophosphate-activated protein kinase; BACH1, BTB domain and CNC homolog 1; EGCG, (-)-Epigallocatechin-3-gallate; ERK, extracellular signal-related kinase; GA, Ginkgolides A; GB, Ginkgolides B; GC, Ginkgolides C; GAS, gastrodin; Keap1, Kelch- like ECH- associated protein 1; LyA, Lyciumamide A; Maf, musculoaponeurotic fibrosarcoma oncogene homologue; miRNA, Nef, Neferine; Nrf2, Nuclear factor erythroid 2-related factor 2; PCA, protocatechualdehyde; PI3K, phosphoinositide 3-kinases; PKC, protein kinase C; RA, rosmarinic acid; Rg1, Ginsenoside Rg1; Sch A, Schizandrin A; Swe, Swertiamarin; THSWD, Tao Hong Si Wu decoction.

**Figure 15:**
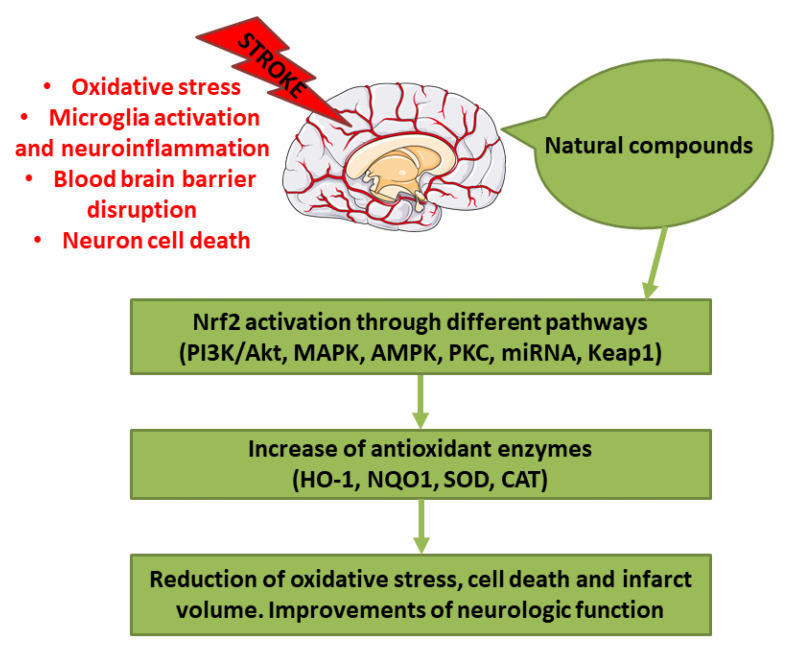
Schematic representation of stroke neurotoxic insults and of the actions exerted by the bioactive compounds. The figure was made taking the images from Servier Medical Art (available at http://smart.servier.com/), licensed under a Creative Commons Attribution 3.0 Unported License (https://creativecommons.org/licenses/by/3.0/). AMPK, monophosphate-activated protein kinase; CAT, catalase; HO-1, Heme oxygenase 1; Keap1, Kelch- like ECH- associated protein 1; MAPK, Mitogen-Activated Protein Kinas; miRNA, microRNA; NQO1, NAD(P)H quinone oxidoreductase 1; PI3K, phosphoinositide 3-kinases; PKC, protein kinase C; SOD, Superoxide dismutase.

A summary of the presented studies, indicating the models used, the doses of the different compounds, the main results and the Nrf2 mechanism is presented in Table 1. Moreover, Figure 14 represents a schema of the different pathways activated by the natural compounds that are involved in Nrf2 modulation. In addition, Figure 15 represents a schematic representation of stroke neurotoxic insults and of the actions exerted by the bioactive compounds.

## 4. Conclusions

Plants represent a rich source of bioactive compounds known for their health beneficial actions, and many of them possess also antioxidant actions, exerted through the activation of the Nrf2 signaling. Interestingly, different natural compounds, owing to their ability to induce Nrf2 activation, showed beneficial actions in experimental stroke models, leading to the increased expression of antioxidant enzymes, including HO-1 and NQO1, and reducing oxidative stress markers. Interestingly, the different bioactive compounds activate Nrf2 modulating different pathways, the most important that are modulated by the different compounds that we described in this review are PI3K/Akt, MAPK, and NF-κB. The members of these signaling pathways, phosphorylating Nfr2 can modulate its action. It is important to notice that some compounds also regulated Nrf2 through miRNA, a type of regulation of gene expression that is gaining more and more importance. However, an important issue to consider is the bioavailability and the amounts of bioactive compounds that can reach the brain. In some cases, these compounds show a low bioavailability and for this reason, the concentrations able to exert therapeutic effects may not be reached in vivo. Indeed, it must be noticed that in some works the concentrations used were higher than those reported in vivo. It could be a good idea to develop new carriers for the delivery of these compounds. In the studies described in this review, some compounds were administered before the induction of the stroke model, while in others after the MCAO. For this reason, it should be important to understand if they can be used as preventive or therapeutic treatment. This is particularly important for stroke, that normally occurs in elderly people and it was shown that aging changes Nrf2 inducibility.

It is important to mention that several bioactive compounds used in combination showed synergistic effects, and their combination showed stronger effects compared to the use of the single compounds. Even if other studies and clinical trials are needed in order to explore the real therapeutic efficacy of these compounds, the use of carriers to reach significant concentrations in the brain, their synergistic effects, and time windows of administration, natural bioactive compounds may be promising to develop new therapeutic strategies against stroke.

## Figures and Tables

**Table 1:** Summary of the experimental studies involving natural compounds able to modulate Nrf2 pathway in in vivo stroke models.

Natural Compound	Sources	Experimental Model	Tested Doses and Administration	Results	Nrf2 Mechanism	Ref.
Theaflavin	Black tea	In vivo: Rats subjected to tMCAO; In vitro: Primary rat NSCs exposed to OGD	In vivo: 10, 50 mg/kg i.v. 2 h after MCAO, daily for a week; In vitro: 2 and 10 µM for 2 h before OGD.	In vivo: ↓ infarct volume and oxidative stress; ↑ memory and learning. In vitro: ↓ apoptosis and oxidative stress; ↑ proliferation.	↓ miRNA-128-3p ↑ Nrf2 and downstream antioxidant enzymes	[28]
(-)-Epigallocatechin-3-gallate	Green tea	Mice subjected to tMCAO	50 mg/kg i.p at the end of surgery and daily for a week	↓ infarct volume; ↑ neurologic function and angiogenesis.	↑ ERK/Nrf2	[30]
(-)-epicatechin	Green tea	WT and Nrf^−/−^ mice subjected to pdMCAO	15 mg/kg by gavage 90 min before pdMCAO	↓ infarct volume	-	[31]
(-)-epicatechin	Green tea	Mouse WT and Nrf^−/−^ astrocytes treated with hemoglobin	10 µM before or after hemoglobin treatment	↓ oxidative stress and AP-1	↑ Nrf2/SOD1	[35]
Nomilin	Citrus fruits	In vivo: Rats subjected to tMCAO; In vitro: SH-SY5Y cells exposed to OGD	In vivo: 50 mg/kg orally 2 h before MCAO (for neurobehavioral tests: 2 h before tests every day for 6 days); In vitro: 0.1, 0.25, 0.5, 1, 2, 4, 8 μM for 12 h after OGD	In vivo: ↓ infarct volume, brain edema, BBB disruption, and oxidative stress; ↑ neurological score. In vitro: ↓ apoptosis and oxidative stress	↑ Nrf2 and antioxidant enzymes	[39]
Naringenin	Citrus fruits	In vivo: Rats subjected to MCAO; In vitro: rat cortical neurons exposed to OGD	In vivo: 80 μM i.p. after MCAO; In vitro: 20, 40, and 80 μM after OGD.	In vivo: ↓ brain edema and apoptosis; ↑ neurological score. In vitro: ↓ apoptosis and oxidative stress; ↑ proliferation.	↑ antioxidant enzymes	[41]
Nobiletin	Citrus peel	Rats subjected to pMCAO	10 and 25 mg/kg i.p. daily starting 3 days before MCAO induction and a dose after	↓ brain edema, infarct volume, oxidative stress, and inflammation; ↑ neurological score.	↑ Nrf2 and antioxidant enzymes	[43]
Linalool	Citrus peel and citrus essential oils	In vivo: rats subjected to tMCAO; In vitro: rat primary astrocyte and microglial cultures treated with glutamate	In vivo: 25 mg/kg intranasal daily for one month In vitro: 100 nM for 24 h	In vivo: ↓ infarct volume and inflammation; ↑ neurological score. In vitro: ↓ inflammation.	-	[46]
S-allyl cysteine	Garlic	In vivo: WT and Nrf2^−/−^ mice subjected to tMCAO; In vitro: rat cortical neurons exposed to OGD	In vivo: 300 mg/kg i.p. 30 min before MCAO; In vitro: 10, 25, and 50 µM pre-treated for 2 h before OGD and also after OGD.	In vivo: ↓ infarct volume, JNK, and p38; ↑ neurological score. In vitro: ↓ JNK and p38; ↑ cell viability.	↑ Nrf2 and downstream antioxidant enzymes	[55]
Diallyl trisulfide	Garlic	In vivo: rats subjected to tMCAO	15 mg/kg, i.p. 4 doses once a day. The first dose 5 min before the onset of reperfusion	↓ brain damage, infarct volume, oxidative stress, and MMP9; ↑ motor function.	↑ Nrf2 and downstream antioxidant enzymes	[56]
Dihydrocapsaicin	Chili peppers	Rats subjected to tMCAO	2.5, 5 and 10 mg/kg i.p. 15 min previous cerebral reperfusion	↓ neurological deficits, infarct area, BBB damage, inflammation, and oxidative stress	↑ Nrf2 and antioxidant enzymes	[57]
Rosmarinic acid	Rosemary and *Lamiaceae* herbs	Mice subjected to tMCAO	10, 20, or 40 mg/kg i.p. at reperfusion	↓ apoptosis, infarct volume, and oxidative stress; ↑ neurological function	↑ PI3K/Akt ↑ Nrf2 and antioxidant enzymes	[58]
Curcumin	Turmeric	Rats subjected to tMCAO	300 mg/kg i.p. 30 min after occlusion.	↓ BBB disruption, brain edema, infarct volume, inflammation, and oxidative stress; ↑ neurological function	↑ Nrf2	[59]
Hexahydrocurcumin	Turmeric	Rats subjected to MCAO	10, 20, and 40 mg/kg i.p. at reperfusion onset	↓ infarct volume, apoptosis inflammation, and oxidative stress; ↑ neurological function	↑ Nrf2 and antioxidant enzymes	[60]
Lyciumamide A	*Lycium barbarum*	In vivo: Rats subjected to tMCAO; In vitro: differentiated SH-SY5Y cells exposed to OGD	In vivo: 40 mg/kg i.p.at the end of MCAO surgery In vitro: 10, 20, 40 µM for 8 h before OGD	In vivo: ↓ infarct volume and oxidative stress; ↑ neurologic function. In vitro: ↓ apoptosis and oxidative stress.	↑ PKCε/Nrf2/antioxidant enzymes	[62]
Mangiferin	Mango and papaya	Rats subjected to tMCAO	25, 50, and 100 mg/kg i.g. daily for 3 days before the MCAO	↓ infarct volume, brain edema, inflammation, and oxidative stress; ↑ neurologic function.	↑ Nrf2 and antioxidant enzymes	[63]
Resveratrol	Grape	In vitro: rat astrocytes In vivo: WT and Nrf2^−/−^ rats subjected to tMCAO	In vitro: 25 μmol/L for 2 h In vivo: 10 mg/kg i.p. 48 h before occlusion	In vivo: ↓ infarct volume. In vitro: ↑ antioxidant enzyme.	↑ Nrf2/NQO1	[65]
Procyanidin B2	Cocoa, apples, grapes	Rats subjected to tMCAO	To evaluate the effect on infarct size and brain edema: 40, 20, or 10 mg/kg i.g 3 h after MCAO. For BBB permeability and other evaluations: 40 mg/kg i.g. once a day, the first 3 h after MCAO. To evaluate neurological function: 40 mg/kg i.g. daily, the first a day after MCAO.	↓ infarct volume, brain edema, BBB disruption, and oxidative stress; ↑ neurologic function.	↑ Nrf2 and downstream antioxidant enzymes	[68]
Myricetin	Vegetables, berries, tea, wine	In vivo: Rats subjected to tMCAO; In vitro: SH-SY5Y cells exposed to OGD	In vivo: 20, 10, 5 mg/kg i.g. 2 h before and every day after MCAO. In vitro: 10, 3.3, 1, 0.33, 0.1 nM for 3 h before OGD	In vivo: ↓ infarct volume and oxidative stress; ↑ neurologic function. In vitro: ↓ apoptosis and oxidative stress.	↑ Nrf2 and antioxidant enzymes	[70]
Chlorogenic acid	Coffea species	Rats subjected to I/R (common carotid arteries occlusion)	In vivo: 500, 100, 20 mg/kg orally	↓ infarct volume, brain edema, apoptosis, and oxidative stress; ↑ neurologic function.	↑ Nrf2 and antioxidant enzymes	[71]
Tocovid	Edible oils	Mice subjected to tMCAO	200 mg/kg orally daily for 1 month as pre-treatment	↓ infarct volume, apoptosis, and oxidative stress	↑ Nrf2	[73]
3H-1,2-dithiole-3-thione	Cruciferous vegetables	In vivo: WT and Nrf2^−/−^ mice subjected to tMCAO; In vitro: mouse microglial cell line BV2 and primary WT and Nrf2^−/−^ microglia treated with LPS	In vivo: 50 mg/kg i.p. 3 h post reperfusion In vitro: 100 µM	↓ infarct volume, brain edema, BBB disruption, immune cell infiltration, microglia activation, and oxidative stress; ↑ neurologic function and survival.	↑ Nrf2/HO-1	[74]
Sulforaphane	Cruciferous vegetables	In vivo: Nrf^−/−^ and WT mice and rats subjected to autologous blood injection In vitro: microglia	In vivo: 5 mg/kg i.p. 30 min and a day after ICH In vitro: 1–10 µM	In vivo: ↑ hematoma clearance; In vitro: ↓ oxidative stress ↑ red blood cell phagocytosis	↑ Nrf2	[75]
Genistein	Soybeans	Ovariectomized rats subjected to tMCAO	10 mg/kg i.p. once a day two weeks before MCAO	↓ infarct volume, neuronal damage, and oxidative stress; ↑ neurologic function.	↑ Nrf2/NQO1	[77]
Biochanin A	Soybeans	Rats subjected to tMCAO	10, 20 and 40 mg/kg i.p. for 2 weeks before MCAO	↓ infarct volume, brain edema, inflammation and oxidative stress; ↑ neurologic function.	↑ Nrf2 and antioxidant enzymes	[78]
Xanthohumol	*Humulus lupulus*	In vivo: Rats subjected to tMCAO; In vitro: rat primary cortical neurons exposed to OGD.	In vivo: 0.4 mg/kg i.p. 10 min before MCAO In vitro: 0.5 μg/mL for 10 min before OGD	In vivo: ↓ infarct volume, neuronal damage, apoptosis, and oxidative stress; ↑ neurologic function and survival rate. In vitro: ↓ apoptosis and oxidative stress	↑ Nrf2 and downstream antioxidant enzymes	[79]
Alpha-lipoic acid	red meat and vegetables	In vivo: Rats subjected to tMCAO; In vitro: rat cortical neurons exposed to OGD	In vivo: 10, 20, 40, and 80 mg/kg i.v. after reperfusion In vitro: 1, 10 and 100 μM for 1 h before 24 h OGD.	In vivo: ↓ infarct volume, brain edema, and oxidative stress; ↑ neurologic function. In vitro: ↓ oxidative stress; ↑ cell viability	↑ Nrf2 and antioxidant enzymes	[81]
Isoquercetin	medicinal and dietary plants	In vivo: Rats subjected to tMCAO; In vitro: primary culture of rat hippocampal neurons exposed to OGD	In vivo: 5, 10, and 20 mg/kg by gavage after MCAO once a day for 3 days;	In vivo: ↓ infarct volume, brain edema, apoptosis, and oxidative stress; ↑ neurologic function. In vitro: ↓ oxidative stress and apoptosis; ↑ cell viability	↑ Nrf2 ↓ NOX4/ROS/NF-κB	[83]
Corilagin	*Phyllanthus emblica*	In vivo: Rats subjected to tMCAO; In vitro: rat primary cortical neurons exposed to OGD	In vivo: 30 mg/kg i.p. once a day for a week, the first 3 h after MCAO. In vitro: 10, 25, and 50 µM pretreatment for 2 h before OGD and for other 24 h after OGD	In vivo: ↓ infarct volume, apoptosis, and oxidative stress; ↑ neurologic function and angiogenesis. In vitro: ↑ cell viability	↑ Nrf2 and antioxidant enzymes	[85]
6″-O-succinylapigenin		In vivo: Rats subjected to tMCAO; In vitro: HT-22 cells exposed to OGD	In vivo: 20, 40, and 60 mg/kg i.p. immediately post occlusion In vitro: 1, 5, or 10 μM 24 h pre-incubation or 5 μM apigenin	In vivo: ↓ infarct volume and oxidative stress; ↑ neurologic function. In vitro: ↑ cell viability	↑ antioxidant enzymes	[86]
Luteoloside	Artichoke and other plants	Rats subjected to tMCAO	20, 40, and 80 mg/kg i.p. immediately and 12 h after MCAO	↓ cerebral edema, infarct volume, and inflammation; ↑ neurologic function.	↑ Nrf2	[89]
Monascin	red yeast rice	Rats subjected to intracerebral 32 hemorrhage model	1, 5, and 10 mg/kg/day i.g. 6 h after ICH and twice a day for 1, 3, or 7 days.	↓ BBB permeability, cerebral edema, and hematoma; ↑ neurologic function.	↑ Nrf2	[90]
Fucoxanthin	Edible brown seaweeds	In vivo: Rats subjected to tMCAO; In vitro: rat primary cortical neurons exposed to OGD	In vivo: 30, 60, and 90 mg/kg i.g. 1 h before MCAO In vitro: 5, 10 and 20 μM before OGD	In vivo: ↓ infarct volume, brain edema, apoptosis, and oxidative stress; ↑ neurologic function. In vitro: ↓ apoptosis and oxidative stress	↑ Nrf2 and antioxidant enzymes	[87]
Korean red ginseng	*Panax ginseng*	WT and Nrf2^−/−^ mice subjected to pdMCAO	100 mg/kg once daily by gavage for 7 days before pdMCAO	↓ infarct volume, reactive astrogliosis ↑ neurologic function	↑ downstream antioxidant enzymes	[92]
Korean red ginseng	*Panax ginseng*	WT and Nrf2^−/−^ mice subjected to pdMCAO	100 mg/kg once a day by gavage for a week before pdMCAO	↓ infarct volume, reactive astrogliosis, and microgliosis	-	[93]
Korean red ginseng	*Panax ginseng*	WT and Nrf2^−/−^ mice subjected to cerebral hypoxia-ischemia (HI)	100 mg/kg orally for a week before HI	↓ neurological deficits, infarct volume, brain edema, inflammation, and reactive gliosis.	↑ Nrf2 and downstream antioxidant enzymes	[94]
Korean red ginseng	*Panax ginseng*	WT and Nrf2^−/−^ mice subjected to cerebral hypoxia-ischemia (HI)	100 mg/kg orally for a week before HI	↓ infarct volume, brain edema, hippocampal CA1 neuronal degeneration, and reactive gliosis.	↑ downstream antioxidant enzymes	[95]
Ginsenoside Rg1	*Panax ginseng*	In vitro: pheochromocytoma PC12 cells exposed to OGD; In vivo: rats subjected to tMCAO	In vitro: 0.01, 0.1, 1, and 10 μM after OGD In vivo: 20 mg/kg	In vivo: ↓ infarct volume and oxidative stress. In vitro: ↓ oxidative stress; ↑ cell viability	↓ miR-144; ↑ Nrf2 and downstream antioxidant enzymes	[96]
Xueshuantong	*Panax notoginseng*	Rats subjected to tMCAO	25, 50, and 100 mg/kg i.p. 1 h after the onset of reperfusion in MCAO rats and for 3 or 7 days.	↓ oxidative stress. ↑ neuronal function and angiogenesis	↑ Nrf2 and downstream antioxidant enzymes	[97]
Protocatechualdehyde	*Salvia miltiorrhiza*	In vivo: Rats subjected to tMCAO; In vitro: differentiated SH-SY5Y cells exposed to OGD	In vivo: 40 mg/kg i.v. 1 h before starting reperfusion In vitro: range 10 to 100 μM for 6 h previous OGD.	In vivo: ↓ infarct volume and oxidative stress. ↑ neurological function. In vitro: ↓ oxidative stress; ↑ cell viability	↑ PKCε/Nrf2/HO-1	[100]
TanshinoneIIA	*Salvia miltiorrhiza*	WT and Nrf2^−/−^ mice subjected to tMCAO	25 mg/kg i.p. 10 min after reperfusion	↓ infarct volume, apoptosis, and oxidative stress. ↑ neurological function.	↑ Nrf2 and downstream antioxidant enzymes	[101]
Salvianolate lyophilized injection	*Salvia miltiorrhiza*	Streptozotocin-induced diabetic rats subjected to tMCAO	5.25, 10.5, and 21 mg/kg i.v. 3 h after tMCAO induction and then daily for 14 days.	↓ neuronal damage, glucose uptake, and inflammation.	↑ Nrf2 and downstream antioxidant enzymes	[102]
Diterpene ginkgolides meglumine injection	*Ginkgo biloba*	In vivo: rats subjected to tMCAO In vitro: PC12 cells exposed to OGD	In vivo: 1, 3, and 10 mg/kg i.v. at the start of reperfusion and 12 h after In vitro: 10 µmol/L for each ginkgolide A, B, or C or 1, 10, and 20 µg/mL DGMI for 24 h after OGD	In vivo: ↓ infarct volume and brain edema. ↑ neurological function. In vitro: ↑ cell viability	↑ Akt/Nrf2	[104]
Ginkgolide A, ginkgolide B, ginkgolide K and bilobalide	*Ginkgo biloba*	In vivo: rats subjected to tMCAO In vitro: SH-SY5Y cells exposed to OGD	In vivo: 1, 2, and 4 mg/kg i.p. 2 h after reperfusion twice a day. In vitro: 25 mg/L after OGD for 6 h	In vivo: ↓ infarct volume. In vitro: ↓ oxidative stress; ↑ cell viability	↑ Akt/Nrf2 and antioxidant enzymes	[105]
Isorhamnetin	*Ginkgo biloba* and other plants	Mice subjected to tMCAO	5 mg/kg i.p. at the starting of reperfusion, and after 24 h	↓ infarct volume, brain edema, apoptosis, BBB disruption, oxidative stress, and inflammation. ↑ sensimotor function.	↑ Nrf2/HO-1	[106]
Tetramethylpyrazine	*Rhizoma Chuanxiong*	Rats subjected to pMCAO	20 mg/kg i.p. 30 min before and an hour after the occlusion.	↓ inflammatory cell infiltration, neuronal loss activation of circulating neutrophils	↑ Nrf2/HO-1	[108]
Z-ligustilide	*Rhizoma Chuanxiong*	Rats subjected to tMCAO	15 mg/kg intranasal route for 3 days before MCAO	↓ infarct volume, brain edema, and BBB disruption. ↑ neurological function.	↑ NQO1	[109]
Senkyunolide I	*Rhizoma Chuanxiong*	Rats subjected to tMCAO	36 and 72 mg/kg i.v. 15 min after MCAO	↓ infarct volume, brain edema, oxidative stress, and apoptosis. ↑ neurological function.	↑ Erk1/2 ↑ Nrf2 and antioxidant enzymes	[110]
Gastrodin	*Gastrodia elata Blume*	Mice subjected to tMCAO	10, 50, and 100 mg/kg i.p. at the starting of cerebral reperfusion	↓ infarct volume, apoptosis, inflammation, and oxidative stress; ↑ neurologic function.	↑ Akt/Nrf2 and downstream antioxidant enzymes	[112]
Gastrodin	*Gastrodia elata Blume*	In vivo: Rats subjected to tMCAO; In vitro: C6 astroglial cells treated with Zn^2+^	In vivo: 20, 40, and 80 mg/kg i.p. at 1 or 6 h after MCAO In vitro: 50, 100, or 250 μM pretreatment or cotreatment	In vivo: ↓ infarct volume; ↑ neurologic function. In vitro: ↓ oxidative stress; ↑ cell viability	↑ Nrf2	[113]
Phenolic components of Gastrodia elata Blume	*Gastrodia elata Blume*	In vivo: rats subjected to tMCAO; In vitro: Primary human astrocytes HA-1800 and SH-SY5Y cells exposed to H_2_O_2_	In vivo: 4 and 40 mg/kg intragastric once per day for a week before MCAO until the sacrifice. In vitro: 15, 25, or 50 μg/mL for 24 h before H_2_O_2_ treatment or 25 μg/mL for 1–48 h before H_2_O_2_	In vivo: ↓ pathological lesions; ↑ motor and cognitive function. In vitro: ↑ cell viability	↑ Nrf2 and downstream antioxidant enzymes	[114]
Gastrodin	*Gastrodia elata Blume*	In vivo: rats subjected to intracerebral hemorrhage model In vitro: rat primary cortical neuron exposed to hemolysate	In vivo: 100 mg/kg i.p. 2 h, a day, and 2 days after surgery In vitro: 10, 100, and 300 µM for 24 h or 100 µM for 0, 12, 24, 48, and 72 h	In vivo: ↓ brain edema, oxidative stress, and apoptosis; ↑ neurological function. In vitro: ↑ cell viability	↑ Nrf2 and downstream antioxidant enzymes	[115]
Schizandrin A	*Schisandra chinensis*	In vivo: rats subjected to tMCAO; In vitro: differentiated SH-SY5Y cells exposed to OGD	In vivo: 20, 40, and 80 mg/kg i.v. before reperfusion In vitro: range 5 to 100 μM for 6 h pretreatment	In vivo: ↓ infarct volume, inflammation, and oxidative stress; ↑ neurological function. In vitro: ↓ inflammation and oxidative stress ↑ cell viability	↑ AMPK/Nrf2 and downstream antioxidant enzymes	[117]
Salidroside	*Rhodiola crenulata*	Rats subjected to tMCAO	15 and 30 mg/kg i.p. once before MCAO and once after reperfusion	↓ infarct volume and oxidative stress; ↑ neurologic function.	↑ Nrf2 and downstream antioxidant enzymes	[119]
Salidroside	*Rhodiola rosea*	Rats subjected to pMCAO	25, 50, and 100 mg/kg i.p. for a week after MCAO	↓ infarct volume and inflammation; ↑ neurologic function.	↑ PI3K/PKB/Nrf2/NFκB	[120]
Total glycosides	*Cistanche deserticola*	Rats subjected to tMCAO	280 mg/kg i.g. daily after MCAO for 2 weeks	↓ infarct volume and oxidative stress; ↑ neurologic function, BBB integrity, angiogenesis, and neuronal remodeling.	↑ Nrf2 and downstream antioxidant enzymes	[121]
Andrographolide	*Andrographis paniculata*	In vitro: primary mouse cerebral endothelial cells exposed to OGD In vivo: rats subjected to tMCAO	In vitro: 10 µM for 6 h before OGD In vivo: 0.1 mg/kg, i.p. immediately after MCAO	In vitro: ↓ cell death In vivo: ↓ free radical formation, BBB disruption, and infarct volume.	↑ p38/Nrf2/HO-1	[122]
Forsythiaside A	*Forsythia suspensa*	Rats subjected to tMCAO	50 mg/kg i.p. for a week after MCAO	↓ apoptosis, endoplasmic reticulum stress, and oxidative stress; ↑ neurologic function, survival rate.	↑ Nrf2 and downstream antioxidant enzymes	[123]
11-Keto-β-boswellic acid	*Boswellia serrata*	In vivo: rats subjected to tMCAO; In vitro: rat primary astrocytes exposed to OGD	In vivo: 25 mg/kg i.p. 1 h after reperfusion In vitro: 10, 30, and 50 µM for 24 h after OGD	In vivo: ↓ infarct volume, apoptosis, and oxidative stress. ↑ neurologic function In vitro: ↓ cell death and oxidative stress	↑ Nrf2 and downstream antioxidant enzymes	[124]
Swertiamarin	*Gentiana macrophylla Pall*	In vivo: mice subjected to tMCAO; In vitro: rat primary hippocampal neurons exposed to OGD	In vivo: 25, 100, and 400 mg/kg i.p. daily for a week before MCAO In vitro: 0.1, 1, and 10 µM for 24 h after OGD	In vivo: ↓ infarct volume, apoptosis, and oxidative stress. ↑ neurologic function In vitro: ↓ cell death and oxidative stress	↑ Nrf2 and downstream antioxidant enzymes	[125]
Neferine	*Nelumbo nucifera Gaertn*	In vivo: rats subjected to pMCAO; In vitro: PC12 cells exposed to tert-butyl hydroperoxide	In vivo: 12.5, 25, and 50 mg/kg i.g. In vitro: 1–10 µM for 24 h as pre- or post-treatment;	In vivo: ↓ infarct volume, oxidative stress and mitochondrial dysfunction. ↑ neurologic function In vitro: ↓ cell death, mitochondrial dysfunction, and oxidative stress	p62/Keap1/Nrf2	[126]
Totarol	*Podocarpus totara*	In vivo: rats subjected to tMCAO; In vitro: primary rat cerebellar granule cells and cortical neurons exposed to OGD or glutamate	In vivo: 0.1, 1, and 10 µg/kg i.v. at 2 h, 4 h and 6 h after MCAO In vitro: 5 µM pretreatment for 24 h	In vivo: ↓ infarct volume and oxidative stress. ↑ neurologic function In vitro: ↓ neurotoxicity and oxidative stress	↑ Akt and downstream antioxidant enzymes	[127]
Leonurine	*Herba leonuri*	WT and Nrf2^−/−^ mice subjected to pMCAO	5, 10, and 15 mg/kg 2 h i.p. after pMCAO	↓ infarct volume and oxidative stress. ↑ neurologic function	↑ Nrf2 and downstream antioxidant enzymes	[128]
Britanin	*Inula lineariifolia*	In vivo: rats subjected to tMCAO In vitro: rat cortical neurons exposed to OGD	In vivo: 25 and 50 mg/kg i.g. at the start of MCAO and dosed twice after reperfusion for 8 h; 25 mg/kg once at 2 h before occlusion, at the onset of occlusion, at reperfusion or 4 h after reperfusion In vitro: 1, 2.5, and 5 µM for 6 h before or after OGD	In vivo: ↓ infarct volume and oxidative stress. ↑ neurologic function In vitro: ↓ neurotoxicity and oxidative stress	↓ Keap1-mediated ubiquitination of Nrf2 ↑ Nrf2 and downstream antioxidant enzymes	[129]
Osthole	*Cnidium monnieri*	In vivo: Mice subjected to global cerebral ischemia In vitro: HT22 murine hippocampal neuronal cells	In vivo: 25, 50, and 100 mg/kg i.p. 30 min before ischemia and after reperfusion In vitro: 25, 50, and 100 µM for 24 h	In vivo: ↓ BBB disruption and oxidative stress. ↑ cognitive function In vitro: ↑ Nrf2	↑ Nrf2	[130]
Trilobatin	*Lithocarpus polystachyus*	In vivo: Rats subjected to tMCAO; In vitro: Primary rat astrocytes and cortical neurons exposed to OGD	In vivo: 5, 10, and 20 mg/kg by gavage at reperfusion onset twice a day for 3 days; to evaluate the time window: 20 mg/kg at 1, 2, 3, 4, and 6 h after MCAO. To discover the effect of TLB on functional recovery after MCAO: 5, 10, and 20 mg/kg at the onset reperfusion twice daily for 28 days after MCAO In vitro: astrocytes: 12.5, 25, 50 μM for 48 h after OGD. Neurons: 6.25, 12.5, 25, 50 μM for 24 h after OGD.	In vivo: ↓ cerebral edema, infarct volume, inflammation, and oxidative stress. ↑ neurological function In vitro: ↓ oxidative stress and inflammation	↑ Nrf2 and downstream antioxidant enzymes	[131]
Achyranthes bidentata polypeptide k	*Achyranthes bidentata Bl.*	BV2 cells exposed to LPS	0.008, 0.04, 0.2, 1, and 5 µg/mL for 30 min before LPS treatment	↓ inflammation	↑ Nrf2/HO-1	[132]
Tryptanthrin	*P. tinctorium*	BV2 cells exposed to LPS	0.1–20 µM for one hour before LPS treatment	↓ inflammation	↑ Nrf2/HO-1	[133]
5,3′-dihydroxy-3,7,4′-trimethoxyflavone	*Siegesbeckia pubescens*	Mouse hippocampal HT22 and microglia BV2 cells exposed respectively to glutamate and LPS	20 µM	↓ oxidative stress and inflammation	↑ Nrf2/HO-1	[134]
Longxuetongluo capsule	*Dracaena cochinchinensis*	BV2 microglial cells exposed to OGD or LPS	0.5, 1 and 2 µg/mL	↑ cell viability; ↓ inflammation	↑ Nrf2/HO-1	[135]
Gualou Guizhi Granule	Composed of 104 compounds	In vivo: Rats subjected to tMCAO; In vitro: PC12 cells exposed H_2_O_2_	In vivo: 3 g/kg i.g. daily for a week after MCAO In vitro: 100, 200, 300 µg/mL for 24 h before H_2_O_2_	In vivo: ↓ oxidative stress In vitro: ↓ oxidative stress	↑ Nrf2/NQO1 and downstream antioxidant enzymes	[136]
Danshensu and hydroxysafflor yellow A (HSYA)	Danshensu: *Salvia miltiorrhiza*. HSYA: *Carthamus tinctorius* L.	In vivo: Rats subjected to tMCAO; In vitro: Primary culture of rat cortical neurons exposed to OGD	In vivo: Danshensu group: 15 mg/kg; HSYA group: 6 mg/kg; Danshensu+HSYA group: 7.5 mg/kg Danshensu plus 3 mg/kg HSYA; i.p. In vitro: 80 μM Danshensu, 80 μM HSYA, and 40 μM Danshensu+40 μM HSYA for 24 h after OGD	In vivo: ↓ infarct volume, apoptosis, inflammation, and oxidative stress; ↑ neurological function In vitro: ↑ cell viability	↑ Nrf2 and downstream antioxidant enzymes	[137]
Safflower extract and aceglutamide	safflower extract: *Carthamus tinctorius*	In vivo: rats subjected to tMCAO; In vitro: differentiated PC12 cells exposed to H_2_O_2_	In vivo: 2.5 mL/kg SAAG; 1.25 g/kg SA; 75 mg/kg AG; i.p. In vitro: 20 µl/mL SAAG; 0.6 mg/mL AG; 10 mg/mL SA; pretreated for 24 h	In vivo: ↓ infarct rate, inflammation, apoptosis, and oxidative stress; ↑ neurologic function. In vitro: ↓ oxidative stress; ↑ cell viability	↑ Nrf2 and downstream antioxidant enzymes	[138]
Salvianolate lyophilized injection and Xueshuantong injection	*Salvia miltiorrhiza* and *Panax notoginsen*, respectively	Rats subjected to tMCAO	SLI: 21 mg/kg; XST: 100 mg/kg; combination: 100 mg/kg XST and 21 mg/kg SLI; i.v. 3 h after MCAO and daily for 3 days	↓ infarct volume, glia activation and oxidative stress; ↑ body weights, neurobehavioral deficits, regional cerebral blood flow	↑ Nrf2 and downstream antioxidant enzymes	[139]
Tao Hong Si Wu decoction	*Radix Rehmanniae Praeparata, Radix Angelicae Sinensis, Rhizoma Ligustici Chuanxiong, Radix Paeoniae Alba, Semen Prunus and Flos Carthami Tinctorii*	Iv vivo: rats subjected to tMCAO; In vitro: PC12 cells exposed to OGD	In vivo: 0.5, 1, and 1.5 mg/kg for 7 days In vitro: 0.25, 0.5, and 1 mg/mL	In vivo: ↓ infarct volume; ↑ neurologic function. In vitro: ↑ cell viability	↑ PI3K/Akt/Nrf2 and downstream antioxidant enzymes	[140]
Berberine, baicalin and jasminoidin	Huang-Lian-Jie-Du-Decoction	Rats subjected to tMCAO	20 mg/kg (Berberine 8.6 mg/kg, baicalin 6.8 mg/kg and jasminoidin 4.6 mg/kg) i.g. for 7 days	↓ infarct volume, oxidative stress, inflammation, and apoptosis; ↑ neurologic function	↑ Nrf2 and downstream antioxidant enzymes	[141]

AP-1, Activator protein 1; BBB, blood brain barrier; DGMI, Diterpene ginkgolides meglumine injection; ERK, extracellular signal-related kinase; H_2_O_2_, Hydrogen peroxide; HO-1, Heme oxygenase 1; HSYA, hydroxysafflor yellow A; ICH, Intracerebral hemorrhage; i.g., intragastrically; i.p., intraperitoneal; i.v., intravenous; Keap1, Kelch- like ECH- associated protein 1; LPS, Lipopolysaccharide; MCAO, middle cerebral artery occlusion; miRNA, microRNA; MMP, Metalloproteinase; Nrf2, nuclear factor erythroid 2-related factor 2; NQO1, NAD(P)H quinone oxidoreductase 1; NSCs, neural stem cells; OGD, oxygen glucose deprivation; PKC, Protein Kinase C; PI3K, Phosphoinositide 3-kinases; pMCAO, permanent middle cerebral artery occlusion; pdMCAO, permanent distal middle cerebral artery occlusion; ROS, Reactive Oxygen Species; tMCAO, transient middle cerebral artery occlusion; SOD, Superoxide dismutase; WT, wildtype.

## Abbreviations

4-HNE	4-hydroxynonenal
8-OHdG	8-hydroxy-2′-deoxyguanosine
α-LA	Alpha-lipoic acid
βTrCP	β-transducin repeat-containing protein
ABPPk	Achyranthes bidentata polypeptide k
Ahr	Aryl hydrocarbon receptor
AMPK	Monophosphate-activated protein kinase
AP-1	Activator protein 1
AQP4	Aquaporin 4
ARE	Antioxidant Response Element
BACH1	BTB domain and CNC homolog 1
BDNF	Brain-Derived Neurotrophic Factor
BBB	Blood Brain Barrier
CAT	Catalase
CBP	CREB-binding protein
CECs	Cerebral endothelial cells
CL	Corilagin
CGA	Chlorogenic acid
COX2	Cyclooxygenase 2
CREB	Cyclic AMP-responsive element binding protein
D3T	3H-1,2-dithiole-3-thione
DATS	Diallyl trisulfide
DCFH-DA	Dichlorofluorescin-Diacetate
DGMI	Diterpene ginkgolides meglumine injection
DHC	Dihydrocapsaicin
DMF	Dimethyl fumarate
DTMF	5,3′-dihydroxy-3,7,4′-trimethoxyflavone
EC	(-)-epicatechin
EGCG	(-)-Epigallocatechin-3-gallate
FA	Forsythiaside A
FGF	Fibroblast growth factor
GA	Ginkgolides A
GAS	Gastrodin
GB	Ginkgolides B
GC	Ginkgolides C
GLGZG	Gualou Guizhi granule
GPx	Glutathione peroxidase
GSH	Glutathione
GSK-3β	Glycogen Synthase Kinase-3β
GSSG	Oxidized glutathione
GST	Glutathione S-transferase
H_2_O_2_	Hydrogen peroxide
HHC	Hexahydrocurcumin
HLJDD	Huang-Lian-Jie-Du-Decoction
HO-1	Heme oxygenase 1
HQO-1	NAD(P)H quinine oxidoreductase
HSYA	Hydroxysafflor yellow A
ICAM-1	Intercellular Adhesion Molecule 1
ICH	Intracerebral hemorrhage
IL	Interleukin
iNOS	inducible nitric oxide synthase
I/R	Ischemia/Reperfusion
KBA	11-Keto-β-boswellic acid
Keap1	Kelch- like ECH- associated protein 1
LPS	Lipopolysaccharide
LyA	Lyciumamide A
LTC	Longxuetongluo capsule
MAPK	Mitogen-Activated Protein Kinase
MAPK/ERK	MAPK/extracellular signal-related kinase
MCA	Middle Cerebral Artery
MCAO	MCA Occlusion
MDA	Malondialdehyde
miRNAs	MicroRNAs
MMP	Metalloproteinase
Nar	Naringenin
Nef	Neferine
NF-κB	Nuclear factor kappa-light-chain-enhancer of activated B cells
NGF	Nerve Growth Factor
NO	Nitric oxide
Nob	Nobiletin
NOX	NADPH oxidase
Nom	Nomilin
NQO1	NAD(P)H quinone oxidoreductase 1
Nrf2	Nuclear factor erythroid 2-related factor 2
NSCs	Neural stem cells
O_2_	Oxygen
OGD	Oxygen Glucose Deprivation
PARP-1	Poly [ADP-ribose] polymerase 1
PB	Procyanidin B2
PCA	Protocatechualdehyde
PCGE	phenolic components of Gastrodia elata Blume
PKC	Protein Kinase C
PI3K	Phosphoinositide 3-kinases
pMCAO	Permanent MCAO
pdMCAO	Permanent distal MCAO
PPARγ	Peroxisome proliferator activated receptor gamma
RA	Rosmarinic acid
Rg1	Ginsenoside Rg1
ROS	Reactive Oxygen Species
SAAG	Safflower extract and aceglutamide
SAC	S-allyl cysteine
SAH	Subarachnoid hemorrhage
Sch A	Schizandrin A
SEI	Senkyunolide I
SLI	Salvianolate lyophilized injection
sMAF	small musculoaponeurotic fibrosarcoma oncogene homologue
SOD	Superoxide dismutase
Swe	Swertiamarin
TGs	Total glycosides
THSWD	Tao Hong Si Wu decoction
TLB	Trilobatin
TMP	Tetramethylpyrazine
TNF	Tumor necrosis factor
tPA	Tissue plasminogen activator
TSA	Tanshinone IIA
VEGF	Vascular Endothelial Growth Factor
VEGFR2	Vascular Endothelial Growth Factor Receptor 2
WT	Wildtype
XN	Xanthohumol
XST	Xueshuantong injection
ZO-1	Zonulin 1
